# Hypoxia-inducible factor 2-alpha-dependent induction of amphiregulin dampens myocardial ischemia-reperfusion injury

**DOI:** 10.1038/s41467-018-03105-2

**Published:** 2018-02-26

**Authors:** Michael Koeppen, Jae W. Lee, Seong-Wook Seo, Kelley S. Brodsky, Simone Kreth, Ivana V. Yang, Peter M. Buttrick, Tobias Eckle, Holger K. Eltzschig

**Affiliations:** 1 Department of Anaesthesiology and Intensive Care Medicine, Tübingen University Hospital, Eberhard-Karls University Tübingen, Tübingen, Germany; 2 Department of Anaesthesiology, Ludwig-Maximilians-University, Muenchen, Germany; 3 Department of Anesthesiology, The University of Texas Health Science Center at Houston, McGovern Medical School, Houston, TX USA; 4 Department of Anesthesiology, University of New Mexico School of Medicine, Albuquerque, NM USA; 5 Division of Cardiology, Department of Medicine, University of Colorado School of Medicine, Aurora, CO USA; 6 Division of Pulmonary Science and Critical Care Medicine, Department of Medicine, University of Colorado School of Medicine, Aurora, CO USA; 7 Department of Anesthesiology, University of Colorado School of Medicine, Aurora, CO USA

## Abstract

Myocardial ischemia–reperfusion injury (IRI) leads to the stabilization of the transcription factors hypoxia-inducible factor 1-alpha (HIF1-alpha) and hypoxia-inducible factor 2-alpha (HIF2-alpha). While previous studies implicate HIF1-alpha in cardioprotection, the role of HIF2-alpha remains elusive. Here we show that HIF2-alpha induces the epithelial growth factor amphiregulin (AREG) to elicit cardioprotection in myocardial IRI. Comparing mice with inducible deletion of *Hif1a* or *Hif2a* in cardiac myocytes, we show that loss of Hif2-alpha increases infarct sizes. Microarray studies in genetic models or cultured human cardiac myocytes implicate HIF2-alpha in the myocardial induction of AREG. Likewise, AREG increases in myocardial tissues from patients with ischemic heart disease. Areg deficiency increases myocardial IRI, as does pharmacologic inhibition of Areg signaling. In contrast, treatment with recombinant Areg provides cardioprotection and reconstitutes mice with *Hif2a* deletion. These studies indicate that HIF2-alpha induces myocardial AREG expression in cardiac myocytes, which increases myocardial ischemia tolerance.

## Abstract (web summary)

Myocardial ischemia–reperfusion injury stabilizes the hypoxia-inducible factor HIF2-alpha. Here, the authors show that HIF2-alpha protects the heart from injury via induction of the epidermal growth factor amphiregulin, and that amphiregulin administration is cardioprotective in mice.

## Introduction

Myocardial infarction is among the leading causes of death in the Western countries^1^. It results from the occlusion of a coronary artery by an intracoronary thrombus, thereby preventing blood flow to the metabolically highly active myocardium. The mainstay therapy for acute myocardial ischemia currently focuses on timely reperfusion—for example by placement of an intracoronary stent^1–4^. However, additional therapeutic approaches that render the myocardium more resistant to ischemia are an important area of investigation. Such approaches would contribute to the preservation of myocardium at risk and could potentially improve outcomes of patients suffering acute myocardial ischemia^3,5,6^.

During myocardial ischemia, the supply and demand ratio for metabolites shifts dramatically—in particular for oxygen—thereby causing profound tissue hypoxia^2,7^. Cellular responses to hypoxia lead to stabilization of hypoxia-dependent transcription factors^8–10^. Indeed, previous studies have suggested the transcription factor hypoxia-inducible transcription factors (HIFs) in cardio-adaptive responses. For example, HIFs mediate the cardioprotective response induced by ischemic preconditioning, where short time periods of ischemia treatment reduces myocardial infarct sizes. During ischemic preconditioning HIFs are stabilized^11^, however, mice with partial deletion of *Hif1a* are not protected by ischemic preconditioning^12^. Similarly, HIFs have been implicated in mediating cardioprotection provided by remote ischemic preconditioning, a cardioprotective strategy where treatment of a limb for short time periods of ischemia results in attenuated myocardial infarct sizes^5,13^.

Due to their high metabolic demand, the functional role of myocytes in cardio-adaptive responses during ischemia has been the focus of many studies^4,14^, and several have indirectly suggested myocardial-expressed HIFs in cardioprotection^14^. However, a significant obstacle for the systematic investigation of HIFs in cardioprotection results from the fact that mice with homozygous deletions of HIFs die early during embryogenesis^2,15,16^. To overcome this problem, we generated mice with inducible myocyte-specific deletion of *Hif1a* or *Hif2a*, the two predominant isoforms of HIFs that mediate alteration in genetic programs during inflammatory or ischemic conditions^17–19^. For this purpose, we crossed transgenic mice with a floxed *Hif1a* or *Hif2a* gene with mice expressing Cre-recombinase under the control of a tamoxifen-inducible myocyte-specific promoter (*Hif1a*^*loxP/loxP*^ Myosin-Cre+ and *Hif2a*^*loxP/loxP*^ Myosin-Cre+ mice, respectively).

Exposure of these two mouse strains to IRI revealed a previously unappreciated role in cardioprotection for myocyte-dependent Hif2-alpha via induction of the growth factor amphiregulin (Areg).

## Results

### Myocyte-specific *Hif2a* deletion enhances myocardial IRI

**Fig. 1:**
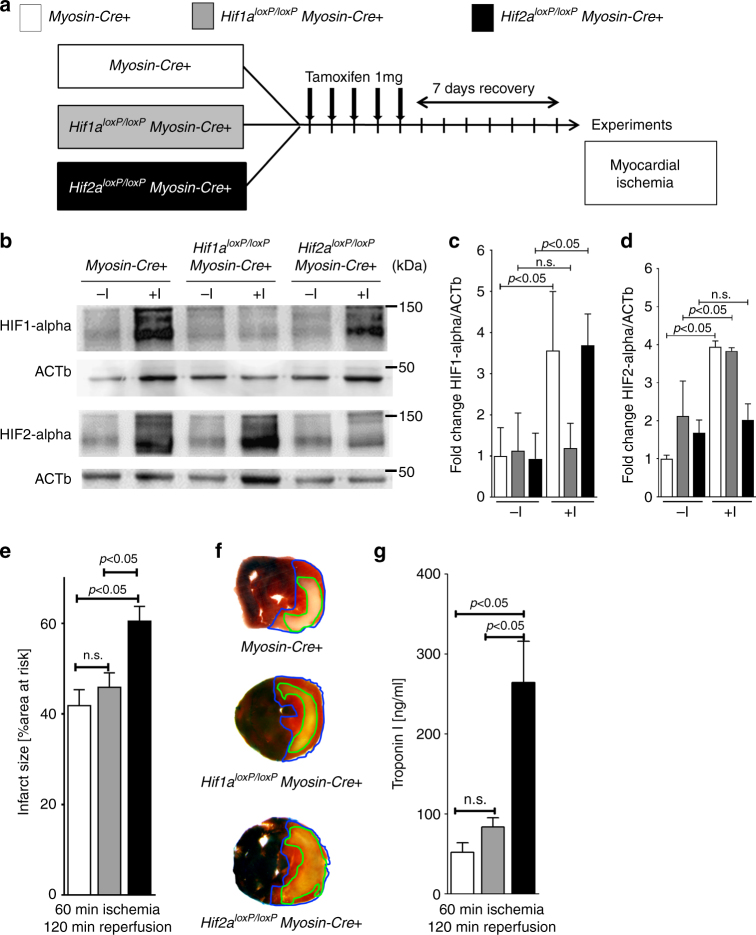
Contribution of myocyte-specific hypoxia-inducible factor (HIF) isoforms Hif1a or Hif2a to cardioprotection. **a** Schematic of breeding approach to generate mice with induced myocyte-specific HIF deletions used in subsequent studies. *Hif1a*^*loxP/loxP*^ or *Hif2a*^*loxP/loxP*^ mice were crossed with Cre-recombinase expressing mice under the control of Myosin-heavy chain promoter (*Myosin-Cre*+); these mice express cre-recombinase under the control of a tamoxifen-inducer. Control animals (*Myosin-Cre*+), *Hif1a*^*loxP/loxP*^*Myosin-Cre*+ or *Hif2a*^*loxP/loxP*^*Myosin-Cre*+ received a daily dose of 1 mg i.p. Tamoxifen five consecutive days to induce the Cre-recombinase activity. After 7 days, animals underwent experimental protocol (60 min of in situ myocardial ischemia followed by 120 min of reperfusion). **b** HIF1-alpha or HIF2-alpha immunoblot analysis from homogenized myocardial tissue, harvested from male *Myosin-Cre*+ and *Hif1a*^*loxP/loxP*^*Myosin-Cre*+ or *Hif2a*^*loxP/loxP*^*Myosin-Cre*+ mice, matched in age and weight. Mice underwent a thoracotomy with no further treatment(-I) or 60 min of myocardial ischemia (+**I**) followed by 120 min reperfusion; β-Actin (ACTb) served as a loading control. One representative blot out of three experiments is shown. **c**, **d** Quantification by densitometry of the HIF-immunoblot results relative to ACTb. Data are expressed as mean fold change ±SD normalized to untreated myocardial tissue from *Myosin-Cre*+ compared by one-way ANOVA followed by Bonferroni’s multiple comparison test (*n* = 3 per group; **c**: F_5,12_ = 6.74,* p* = 0.0033; **d**: F_5,12_ = 21.85, *p* < 0.0001). **e** Infarct sizes ±SD in *Myosin-Cre*+, *Hif1a*^*loxP/loxP*^ *Myosin-Cre+ *or *Hif2a*^*loxP/loxP*^*Myosin*-*Cre+* mice, presented as the percentage to the area-at-risk after 60 min of ischemia, followed by 120 min of reperfusion (*Myosin-Cre*+ *n* = 5; *Hif1a*^*loxP/loxP*^*Myosin-Cre+ n* = 5; *Hif2a*^*loxP/loxP*^*Myosin-Cre+ n* = 4; per group mean ± SD; compared by one-way ANOVA followed by Bonferroni’s multiple comparison test; F_2,11_ = 7.901; *p* = 0.0075). **f** Representative infarct staining from *Myosin-Cre*+, *Hif1a*^*loxP/loxP*^*Myosin-Cre*+ or *Hif2a*^*loxP/loxP*^*Myosin-Cre*+. **g** Troponin serum levels after 60 min ischemia, followed by 120 min of reperfusion in *Myosin-Cre*+*, Hif1a*^*loxP/loxP*^*Myosin-Cre+* or *Hif2a*^*loxP/loxP*^*Myosin-Cre*+ (*Myosin-Cre*+ *n* = 5; *Hif1a*^*loxP/loxP*^*Myosin-Cre*+ *n* = 5, and *Hif2a*^*loxP/loxP*^*Myosin-Cre*+ *n* = 4 per group; presented as mean ± SD; compared by one-way ANOVA followed by Bonferroni’s multiple comparison test; F_2,11_ = 19.14, *p* = 0.0003)

Based on previous studies implicating HIFs in organ-protection during ischemia and reperfusion injury^2,7^, we hypothesized that myocyte-specific HIFs dampen myocardial ischemia and reperfusion injury. To overcome the problem that mice with a global *Hif1a* or *Hif2a* deletion die during embryogenesis^20^, we generated mice with induced deletion of *Hif1a* or *Hif2a* in cardiac myocytes. To achieve this, we crossed *Hif1a*^*loxP/loxP*^ or *Hif2a*^*loxP/loxP*^ mice with mice expressing tamoxifen-inducible Cre-recombinase under the Myosin-heavy chain promoter (*Myosin-Cre*+)^21^. To induce a *Hif1a* or *Hif2a* deletion, respectively, we treated these mice with daily tamoxifen injections for 5 days (1 mg i.p./day), followed by a 7 day recovery period (Fig. 1a). Subsequently, we exposed the mice to in situ myocardial ischemia–reperfusion. Western blot studies for Hif1-alpha or Hif2-alpha showed that protein levels increased following 60 min of ischemia and 120 min of reperfusion in Myosin-Cre+ mice. In contrast, the respective HIF-isoform in the post-ischemic myocardium decreased notably in *Hif1a*- or *Hif2a*-deficient animals (Fig. 1b–d; Supplementary Fig. 1). These findings demonstrate an increase of Hif1-alpha and Hif2-alpha levels following myocardial ischemia and reperfusion injury, while in genetic models for *Hif1a* or *Hif2a* deletion this response is attenuated. These findings suggest that the mouse lines generated allow us to assess the individual function of myocyte-specific Hif1-alpha versus Hif2-alpha during myocardial ischemia and reperfusion injury.

To assess the functional role of cardiac myocyte-specific Hif1-alpha or Hif2-alpha in cardioprotection, we exposed *Hif1a*^*loxP/loxP*^ Myosin-Cre+ of *Hif2a*^*loxP/loxP*^ Myosin-Cre+ mice to myocardial ischemia and reperfusion injury and measured myocardial injury by infarct size area or serum troponin levels. Surprisingly, we found the predominant phenotype in *Hif2a*^*loxP/loxP*^ Myosin-Cre+ mice. Indeed, mice with induced *Hif2a* deletion in cardiac myocytes experienced dramatic increases in myocardial injury when compared to *Hif1a*^*loxP/loxP*^ Myosin-Cre+ mice or Myosin-Cre+ controls (Fig. 1e–g). Together, these studies suggest a role for myocyte-specific Hif2-alpha in cardioprotection during ischemia and reperfusion injury.

### Identification of Areg as Hif2-alpha target during IRI

**Fig. 2:**
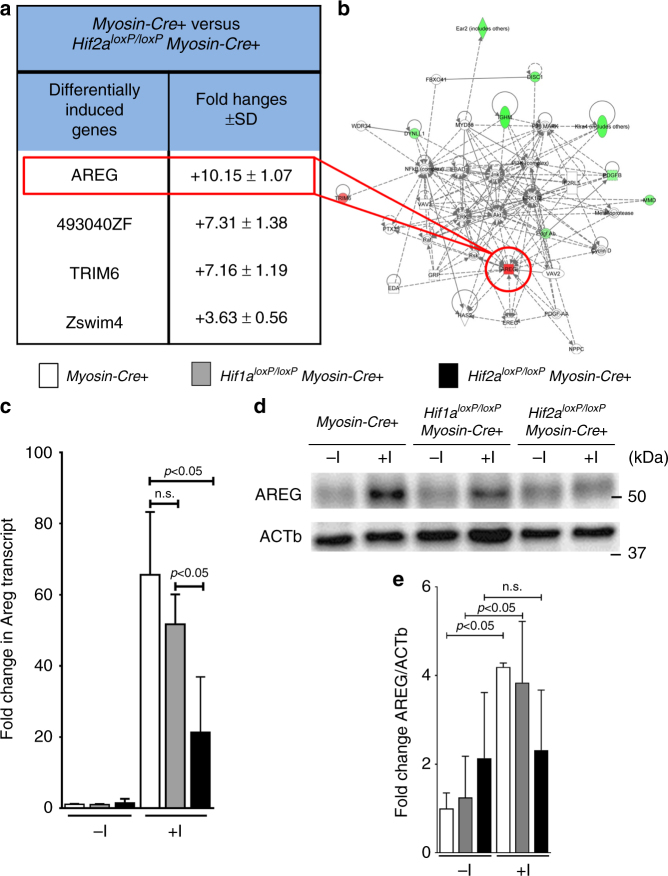
Hif2a-dependent induction of amphiregulin during myocardial injury. **a**, **b** *Myosin-Cre*+ or *Hif2a*^*loxP/loxP*^*Myosin-Cre*+ underwent 45 min myocardial ischemia, followed by 120 min reperfusion. After harvest of the area-at-risk, total RNA was probed for transcriptional changes using a microarray technique (Agilent-014868 Whole Mouse Genome Microarray 4x44K G4122F Probe Name version). Transcriptional changes were calculated in relation to the myocardial baseline expression in untreated littermate controls of the same genotype. Array data have been deposited at http://www.ncbi.nlm.nih.gov/geo/ (accession number GSE67308). **a** List of “top hits” of genes transcriptionally induced in the Area-At-Risk in *Myosin-Cre*+ but unchanged in *Hif2a*^*loxP/loxP*^*Myosin-Cre*+ (*n* = 4 per group, mean ± SD). Note: the most prominent differential regulation was found for the epidermal growth factor amphiregulin (Areg). **b** Analysis of relevant networks in the IPA (*Ingenuity Pathway Analysis*^*®*^) library most significant to the dataset. Note: selective induction of Areg **c** *Myosin-Cre*+, *Hif1a*^*loxP/loxP*^*Myosin-Cre*+, or *Hif2a*^*loxP/loxP*^*Myosin-Cre*+ underwent 60 min myocardial ischemia, following 120 min reperfusion. Total RNA from the area-at-risk was probed for transcriptional changes of *Areg* by real-time RT-PCR. Transcriptional changes were calculated relative to an internal housekeeping gene (*Actb*). Data are expressed as mean fold change ± SD compared to untreated myocardial tissue from littermates of the respective genotype compared by one-way ANOVA followed by Bonferroni’s multiple comparison test (*Myosin-Cre*+: −I *n* = 6, +I *n* = 5; *Hif1a*^*loxP/loxP*^*Myosin-Cre*+: −I *n* = 3; +I *n* = 4; *Hif2a*^*loxP/loxP*^*Myosin-Cre*+: −I *n* = 6; +I *n* = 7; F_5,25_ = 33.53; *p* < 0.0001). **d** AREG immunoblot performed for protein isolated from the ischemic myocardial tissue after 60 min of ischemia and 120 min reperfusion or untreated littermate controls of *Myosin-Cre*+*, Hif1a*^*loxP/loxP*^*Myosin-Cre*+ or *Hif2a*^*loxP/loxP*^*Myosin-Cre*+, respectively. A representative image of three individual experiments is presented. **e** Quantification by densitometry of the AREG immunoblot results relative to ACTb. Data are expressed as mean fold change ± SD normalized to untreated myocardial tissue from *Myosin-Cre*+ compared by one-way ANOVA followed by Bonferroni’s multiple comparison test (*n* = 3 per group; F_5,12_ = 4.487; *p* = 0.0154)

After having shown that mice with induced *Hif2a* deletion in cardiac myocytes experience increased myocardial injury, we next investigated a transcriptional mechanism that could elicit Hif2a-dependent cardioprotection. Previous studies implied Hif1-alpha in cardioprotection from ischemia^12–14^. In contrast, a functional role of Hif2-alpha in cardioprotection is largely unknown. Since HIFs mediate adaptive responses through the induction of target genes, we performed a microarray study comparing transcript levels of the post-ischemic myocardium from *Myosin-Cre*+ or *Hif2a*^*loxP/loxP*^ *Myosin-Cre+* mice. Consistent with a role of myocyte-specific Hif2-alpha as a transcriptional inducer, we identified a set of genes that increased in the post-ischemic myocardium of *Myosin-Cre*+ but not in *Hif2a*^*loxP/loxP*^*Myosin-Cre+* mice. Among transcript levels upregulated in an Hif2-alpha-dependent fashion, the strongest differentially regulated transcript was the epithelial growth factor Amphiregulin (Areg) (Fig. 2a, b). To follow-up on the findings from the microarray study, we examined Areg transcript and protein levels in the area-at-risk (AAR) of *Hif1a*^*loxP/loxP*^ *Myosin-Cre*+, *Hif2a*^*loxP/loxP*^ *Myosin-Cre+*, or *Myosin-Cre+* mice. Consistent with our findings in the microarray, we observed that Areg transcript and protein levels significantly increased in *Myosin-Cre*+ and mice with cardiomyocyte-specific *Hif1a* deficiency. In contrast, the induction of Areg in post-ischemic myocardial tissues was significantly attenuated in *Hif2a*^*loxP/loxP*^ *Myosin-Cre*+ mice (Fig. 2c–e; Supplementary Fig. 2). Together with previous studies from the cancer field suggesting that Hif2-alpha binds to the *Areg* promoter and induces its transcription^22,23^ our findings indicate that Hif2-alpha transcriptionally induces Areg during murine myocardial IRI.

### HIF2-alpha regulates AREG expression in human cardiac myocytes

**Fig. 3:**
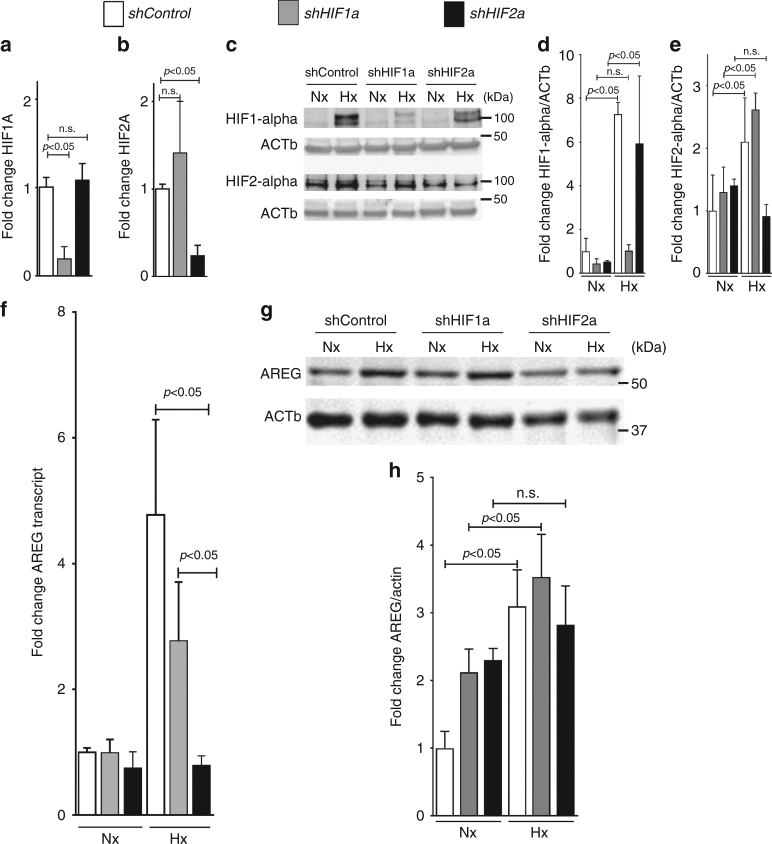
Functional role of HIF2A in the transcriptional regulation of amphiregulin (AREG) in human cardiac myocytes. **a–e** Human cardiac myocytes (HCM) underwent control, HIF1A-specific or HIF2a-specific short hairpin RNA (shRNA) lentiviral transfection to suppress transcription of hypoxia-inducible factors HIF1A or HIF2A. **a**, **b** shRNA-transfected HCM were exposed to ambient hypoxia (1% oxygen) for 16 h and analysis of transcript changes by RT-PCR of HIF1A or HIF2A, respectively. Transcriptional changes were calculated relative to an internal housekeeping gene (Actin-b). Data are expressed as mean fold change ± SD compared to normoxic cells (*n* = 6 per group). **c** Immunoblot for HIF1A or HIF2A from shRNA-transfected normoxic or hypoxic HCM. β-Actin (ACTb) served as a loading control. **d**, **e** Quantification by densitometry of the HIF1-alpha or HIF2-alpha immunoblot results relative to ACTb. Data are expressed as mean fold change ± SD normalized to *shControl* compared by one-way ANOVA followed by Bonferroni’s multiple comparison test (*n* = 3 per group; **d** F_5,11_ = 14.48, *p* = 0.0002; **e** F_5,11_ = 6,726, *p* = 0.0042). **f** HCM transfected with shRNA directed against *HIF1A*, *HIF2A* or control shRNA were exposed to ambient hypoxia (1% oxygen) for 16 h or were maintained under normoxic conditions (21% oxygen). Subsequently, total RNA was isolated and probed by RT-PCR for transcriptional changes of amphiregulin (*AREG*). Data are expressed as mean fold change ± SD compared to normoxic cells (*n* = 3 per group). Transcriptional changes were calculated relative to an internal housekeeping gene (*ACTb*). Data compared by one-way ANOVA followed by Bonferroni’s multiple comparison test (F_5,12_ = 14.33;* p = 0.0001*). **g** HCM transfected with shRNA directed against *HIF1A*, *HIF2A,* or control shRNA, total protein was isolated and immunoblotted for AREG. b-Actin (Actb) served as a loading control. One representative blot out of three experiments is shown. **h** Quantification by densitometry of the HIF immunoblot results relative to ACTb. Data are expressed as mean fold change ± SD normalized to normoxic cells (*shControl)* and compared by one-way ANOVA followed by Bonferroni’s multiple comparison test (*n* = 3 per group; F_5,12_ = 11, 58; *p* = 0.0003)

Based on the findings that Hif2-alpha regulated Areg transcript and protein levels in a murine in vivo model of myocardial ischemia and reperfusion injury, we next performed studies in cultured human cardiomyocytes to demonstrate that the observed changes in murine Areg levels also occur in human myocardial tissues. Thus, we exposed human cardiac myocytes (HCM) to hypoxia and measured AREG transcript and protein levels. To address a functional role for HIF2-alpha in AREG induction, we generated HCM cells lines with stable lentiviral-mediated shRNA-mediated repression of HIF1-alpha or HIF2-alpha. Indeed, lentiviral-mediated shRNA transduction in HCM repressed HIF1-alpha or HIF2-alpha transcript levels at baseline (Fig. 3a, b). To examine if the lentiviral-mediated shRNA repression of HIF1-alpha or HIF2-alpha also attenuated HIF protein stabilization during hypoxia, we exposed control-transduced HCMs, or HCMs transduced with lentiviral shRNA targeting HIF1-alpha or HIF2-alpha to ambient hypoxia (1% oxygen for 16 h). While exposure of control cells to ambient hypoxia stabilized HIF1-alpha and HIF2-alpha protein, these responses were abolished in HCM cell lines with corresponding shRNA-mediated HIF repression (Fig. 3c–e). Together, these studies indicate that these human myocyte cell lines can be used to study differential roles for HIF1-alpha- or HIF2-alpha-dependent gene induction during hypoxia. Indeed, induction of AREG transcript and protein levels remained intact in HCM transduced with control or HIF1-alpha-repressing shRNA, but were entirely abolished following shRNA-mediated repression of HIF2-alpha (Fig. 3f–h; Supplementary Fig. 3). Taken together, these findings demonstrate that HIF2-alpha is a transcriptional driver of *AREG* in HCM.

### AREG levels increase in patients with ischemic heart disease

**Fig. 4:**
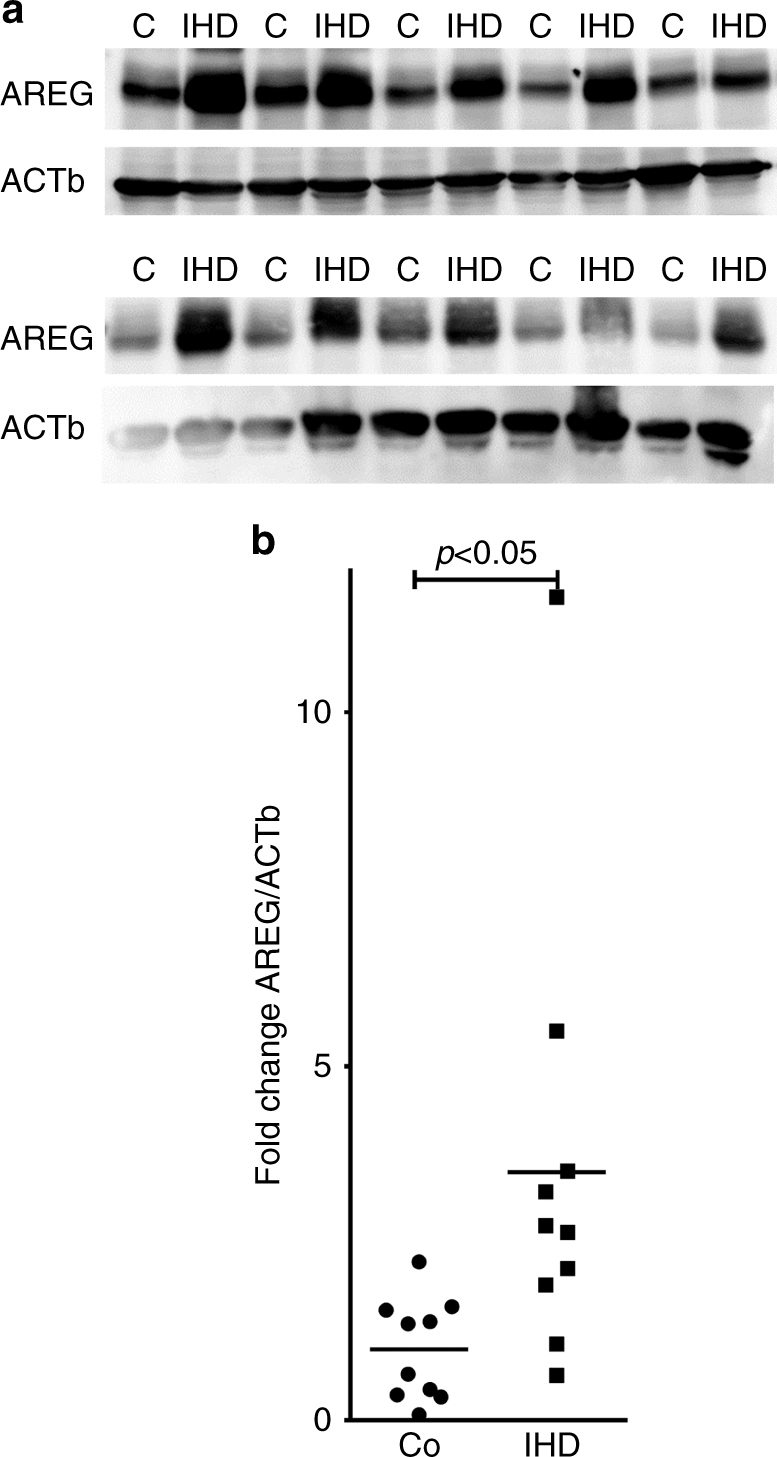
Cardiac amphiregulin protein levels in myocardial tissues from healthy controls or patients with ischemic heart disease (IHD). **a** Amphiregulin (AREG) protein levels were determined by immunoblot after total protein isolation from control, healthy myocardial tissue (labeled C), or samples from patients with ischemic heart disease (labeled IHD). β-Actin (ACTb) served as a loading control. **b** Quantification by densitometry of the AREG immunoblot results relative to ACTb (*n* = 10 in both groups; data compared by two-sided Mann–Whitney test)

After having shown that Areg levels increased in a murine myocardial ischemia and reperfusion injury model or in HCM exposed to hypoxia, we next performed proof-of-principle studies to examine AREG levels in cardiac tissues obtained from healthy controls or patients with ischemic heart disease (IHD). We received these cardiac tissues from a human biobank that stores human heart samples^14^. Cardiac tissues from IHD patients were retrieved from explanted hearts during cardiac transplantation. Cardiac tissues that served as healthy controls were taken from donor hearts that were deemed sufficient as a cardiac allograft, but could not be used for transplantation due to logistic reasons. Consistent with a functional role for myocardial ischemia in AREG induction, AREG protein levels were elevated in patients with IHD compared to healthy controls (Fig. 4a, b; Supplementary Fig. 4). Taken together, these proof-of-principle studies indicate that AREG expression increased in cardiac tissues from patients experiencing myocardial ischemia.

### Areg deficiency is associated with enlarged infarct sizes

**Fig. 5:**
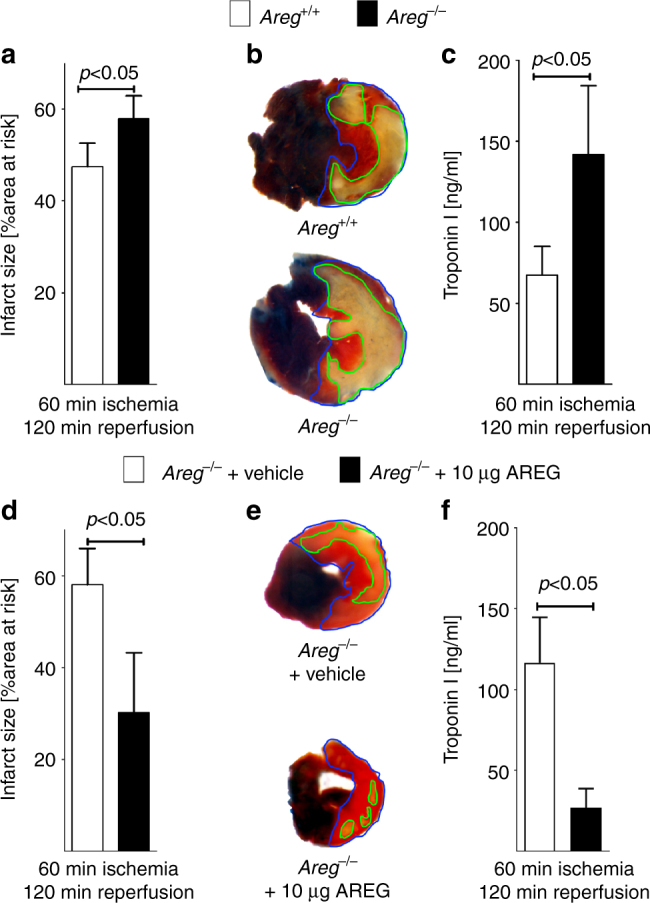
Myocardial tissue injury in *Areg*^*−/−*^ mice exposed to myocardial ischemia and reperfusion injury. **a**–**f** Experimental animals were exposed to 60 min of myocardial ischemia, followed by 120 min of reperfusion; infarct sizes were measured by double staining with Evan’s blue and triphenyltetrazolium chloride and serum samples were collected. All infarct sizes are presented as the percentage of infarcted tissue in relation to the area-at-risk. Serum troponin levels were determined by ELISA (**a**). Infarct sizes were determined in gene-targeted mice for amphiregulin (*Areg*^*−/−*^ mice) or isogenic control animals (*Areg*^*+/+*^ mice) (*n* = 5 per group). **b** Representative infarct staining from *Areg*^*−/−*^ or *Areg*^*+/+*^. **c** Troponin serum levels in *Areg*^*−/−*^ or *Areg*^*+/+*^ mice (*n* = 5 per group). **d** Reconstitution of *Areg*^*−/−*^ mice with recombinant AREG. Infarct sizes in *Areg*^*−/−*^ treated with vehicle or 10 μg of recombinant murine Areg, administered over 15 min period by an indwelling catheter (*n* = 6 per group). **e** Representative infarct staining from *Areg*^*−/−*^ mice treated with vehicle or 10 µg recombinant murine Areg. **f** Troponin serum levels after myocardial IR injury of *Areg*^*−/−*^ mice treated with vehicle or 10 µg recombinant murine Areg via an intraarterial catheter over a 15 min period (*n* = 7 per group). All data presented as mean ± SD. Statistical significance assessed by two-sided, unpaired Student’s *t*-test

After having shown that murine and human myocardial AREG levels increased during conditions of limited oxygen availability via transcriptional control by HIF2-alpha, we addressed the functional role of AREG during IRI. Since mice with induced *Hif2a* deletion in cardiac myocytes experienced larger myocardial injury, we anticipated a cardioprotective role of Areg. To address this hypothesis, we performed studies in previously described mice gene-targeted for *Areg*^24^. Exposure of *Areg*^*−/−*^ mice to 60 min of myocardial ischemia followed by 120 min of reperfusion revealed larger infarct sizes and elevated levels of the myocardial necrosis marker troponin I (Fig. 5a–c). Moreover, reconstitution of *Areg*^*−/−*^ mice via intravascular infusion of murine recombinant Areg reduced ischemic myocardial tissue injury, including smaller infarct sizes (Fig. 5d, e) and attenuated release of troponin I (Fig. 5f). Together, these findings suggest that genetic deletion of *Areg* increases myocardial tissue injury following murine ischemia and reperfusion injury, while reconstitution with 10 µg recombinant Areg can rescue the phenotype of *Areg*^*−/−*^ mice.

### Blockade of ErbB1 signaling increases myocardial injury

**Fig. 6:**
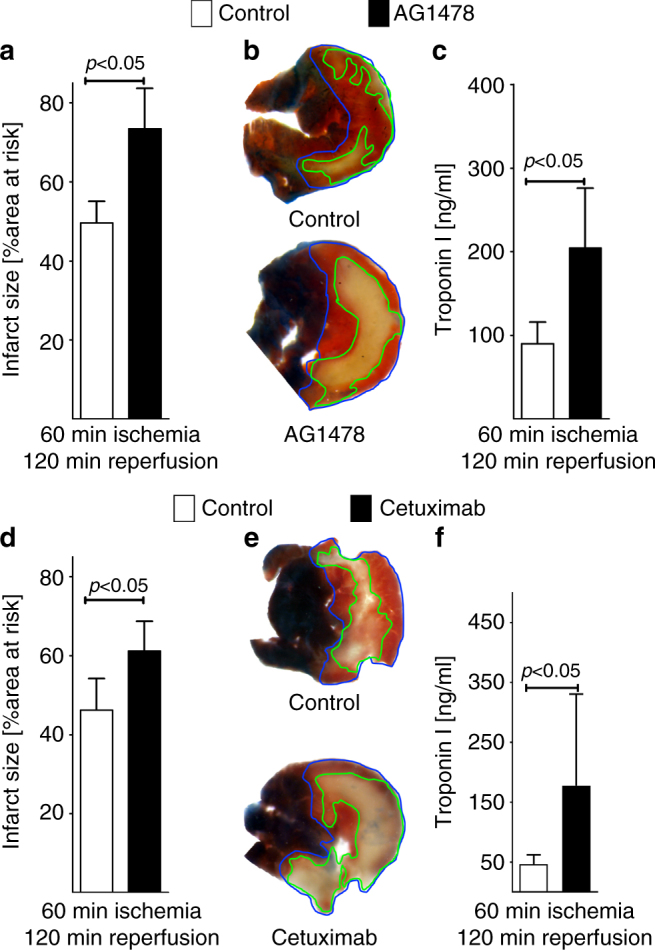
Pharmacologic inhibition of the amphiregulin receptor ErbB1 during murine myocardial ischemia–reperfusion injury. **a**–**c** Mice underwent 60 min of myocardial ischemia, followed by 120 min of reperfusion; infarct sizes were measured by double staining with Evan’s blue and triphenyltetrazolium chloride and serum samples were collected. All infarct sizes are presented as the percentage of infarcted tissue in relation to the area-at-risk. Serum Troponin levels were determined by ELISA. **a** Infarct sizes of C57/BL6 mice after 60 min of ischemia and 120 min reperfusion, treated prior to myocardial ischemia reperfusion with vehicle or an ERBB1-specific inhibitor (AG1478), administered 15 min prior to ischemia via an indwelling arterial catheter. Data presented as the percentage of infarcted area in relation to area-at-risk (*n* = 4 per group). **b** Representative infarct staining of C57/BL6 treated with vehicle or AG1478. **c** Troponin serum levels of C57/BL6 mice, after 60 min ischemia, 120 min reperfusion, treated with vehicle or AG1478 (*n* = 5 per group). **d** Infarct sizes of C57/BL6 mice after 60 min of ischemia and 120 min reperfusion, treated 15 min prior to ischemia via an indwelling arterial catheter with vehicle or a highly specific ERBB1-specific inhibitor (Cetuximab). Data presented as the percentage of infarcted area in relation to area-at-risk (control *n* = 4; cetuximab *n* = 8). **e** Representative infarct staining of C57/BL6 treated with vehicle or Cetuximab. **f** Troponin serum levels of C57/BL6 mice after 60 min ischemia, 120 min reperfusion, treated with vehicle or Cetuximab (*n* = 7 per group). All data in this figure is presented as mean ± SD. Statistical significance assessed by two-sided, unpaired Student’s *t*-test

Since genetic deletion of *Areg* profoundly increased myocardial injury, we performed pharmacologic studies in wild-type mice targeting the Areg receptor ErbB1. Indeed, cardiac tissues express receptors for epidermal growth factors (ErbB receptors), which play important roles in cardiac development^25^. ErbB1 is expressed on cardiac myocytes, but undetectable on vascular endothelia^26^ and previous studies demonstrate that AREG solely binds and signals through the ErbB1^27^. Based on these studies, we hypothesized that AREG elicits its cardioprotective effects via activation of the ErbB1 receptor. To address this hypothesis, we performed myocardial ischemia and reperfusion injury in the presence of the pharmacologic ErbB1 receptor antagonist AG1478^28^. Mice received 20 mmol/kg of AG1478 or vehicle 30 min prior to myocardial ischemia, given as slow infusion via a catheter placed into the carotid artery. Subsequently, mice underwent 60 min of myocardial ischemia and 120 min of reperfusion. Consistent with a functional role for AREG in cardioprotection, treatment with the ErbB1 receptor antagonist AG1478 increased myocardial infarct sizes and significantly elevated serum troponin levels (Fig. 6a–c)—a phenotype similar to the findings in *Hif2a*^*loxP/loxP*^ *Myosin-Cre+* or *Areg*^*−/−*^ mice. Together these findings indicate that AREG-dependent signaling events through the ErbB1 receptor promote cardioprotection from acute myocardial ischemia and reperfusion injury.

### Inhibition of ErbB1-ligand binding increases infarct sizes

As next step, we sought to confirm the above findings with the pharmacologic ErbB1 inhibitor AG1478 utilizing a more specific approach for the ErB1 receptor. For this purpose, we used the recombinant, monoclonal antibody cetuximab, which binds solely to the extracellular domain of the ErbB1. Binding of cetuximab to ErbB1 completely inhibits binding of the natural ligand and activation of the receptor signaling cascade^29–32^. For the purpose of this study, mice received 20 mg/kg of cetuximab or vehicle 15 min prior to myocardial ischemia, given as slow infusion via a carotid artery catheter. Subsequently, mice were submitted to 60 min of myocardial ischemia followed by 120 min of reperfusion. Consistent with the effect of pharmacological inhibition of ErbB1 utilizing AG1478, treatment with the anti-ErbB1 antibody cetuximab was associated with increased myocardial infarct sizes and significantly elevated serum troponin levels (Fig. 6d–f). Together these findings indicate that ErbB1 signaling events promote cardioprotection from acute myocardial ischemia and reperfusion injury.

### Recombinant Areg treatment dampens murine IRI

**Fig. 7:**
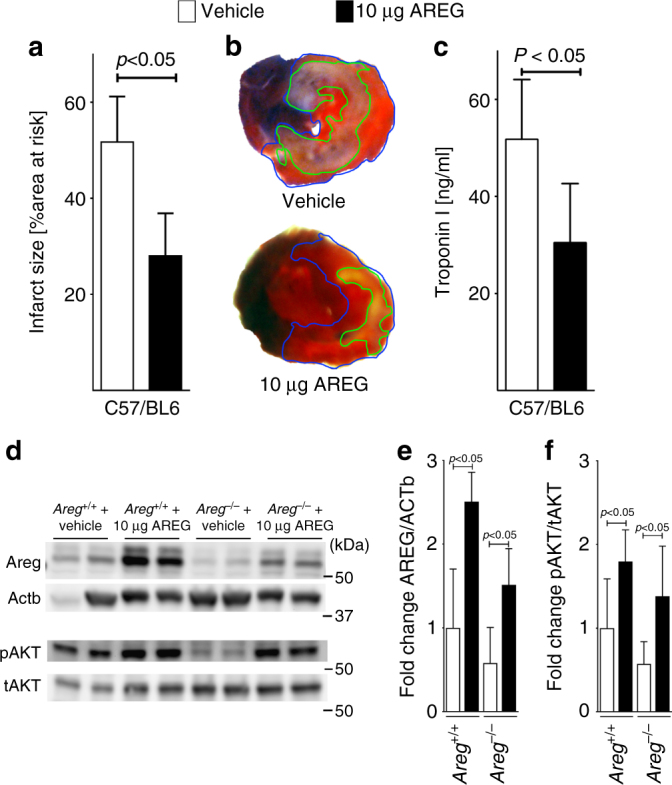
Treatment of murine myocardial ischemia and reperfusion injury with recombinant amphiregulin. **a**–**c** Wild-type were exposed to 60 min of myocardial ischemia, followed by 120 min of reperfusion; infarct sizes were measured by double staining with Evan’s blue and triphenyltetrazolium chloride and serum samples were collected. All Infarct sizes are presented as the percentage of infarcted tissue in relation to the area-at-risk. Serum troponin levels were determined by ELISA. **a** Infarct sizes of C57/BL6 mice after 60 min of ischemia and 120 min reperfusion, treated with vehicle or 10 µg recombinant murine AREG, administered over a 15 min period via an indwelling arterial catheter. Data presented are the percentage of infarcted area in relation to area-at-risk. Statistical significance assessed by two-sided, unpaired Student’s *t*-test (*n* = 7 per group; data presented as mean ± SD). **b** Representative infarct staining of C57/BL6 treated with vehicle or 10 µg recombinant murine AREG. **c** Serum troponin levels of C57/BL6 mice, after 60 min ischemia, 120 min reperfusion, treated with vehicle or 10 µg recombinant murine Areg. Statistical significance assessed by two-sided, unpaired Student’s *t*-test (*n* = 5 per group; data presented as mean ± SD). **d** *Areg*^*+/+*^ or *Areg*^*−/−*^ mice received treatment with vehicle or 10 μg of recombinant murine AREG, administered over a 15 min period by an indwelling catheter. The animals underwent 60 min ischemia and 120 min reperfusion, followed by total protein isolation form the area-at-risk. Upper panel: protein was immunoblotted for AREG, β-actin (ACTb) served as a loading control. Lower panel: protein was immunoblotted for total AKT (tAKT) and phosphorylated AKT (pAKT), respectively. Two samples for each condition and genotype are presented. One representative blot out of three experiments is shown. **e**, **f** Quantification by densitometry of the AREG and pAKT immunoblot results relative to ACTb or Total AKT (tAKT). Data are expressed as mean fold change ± SD normalized to vehicle treated *Areg*^*+/+*^ and compared by one-way ANOVA followed by Bonferroni’s multiple comparison test (*Areg*^*+/+*^+Vehicle *n* = 5; all other groups *n* = 6 mice per group, **e** F_3,19_ = 23,11, *p* < 0.0001; **f** F_3,20_ = 9,549, *p* = 0.0002)

Based on our studies in mice with induced *Hif2a* deletion in cardiac myocytes or studies in mice with global *Areg* deletion, we hypothesized that treatment with recombinant murine Areg could provide cardioprotection from ischemia and reperfusion injury. We used a treatment regime consistent of 10 µg recombinant Areg given as a slow infusion via a catheter placed into the carotid artery 15 min prior to the onset of myocardial ischemia. Control mice received a similar infusion with vehicle. Subsequent exposure to 60 min of myocardial ischemia followed by 120 min of reperfusion was associated with significantly reduced myocardial infarct sizes and troponin I serum levels compared to vehicle treated mice (Fig. 7a–c; Supplementary Fig. 5).

Next, we investigated whether intraarterial administration of recombinant murine Areg increases myocardial Areg protein levels. For this purpose, we treated mice with recombinant Areg and submitted them to our model of myocardial ischemia and reperfusion injury. As controls we used *Areg*^*+/+*^ or *Areg*^*−/−*^ animals receiving vehicle infusion. After 120 min of reperfusion, we identified the AAR by Evans blue counterstain technique and immunoblotted for murine Areg protein. Myocardial ischemia and reperfusion alone increased myocardial Areg levels (Fig. 7d, *Areg*^*+/+*^ +Vehicle). Treatment with recombinant murine Areg was associated with dramatically increased Areg protein levels in wild-type or in *Areg*^*−/−*^ mice (Fig. 7d, upper panel and Fig. 7e). Together, these findings confirm that exogenous administration of recombinant Areg leads to elevated Areg protein levels in the myocardium, and provides a rational for the therapeutic effects observed following treatment with recombinant Areg.

After having shown that treatment with recombinant Areg reduced myocardial injury following ischemia and reperfusion, we next investigated whether administration of recombinant murine Areg activates cardioprotective pathway, e.g. via activation of survival kinases such as Akt^33^. As described above, we treated *Areg*^*+/+*^ and *Areg*^*−/−*^ mice with recombinant Areg, and immunoblotted for Akt (tAkt) and the activated form (phosphorylated Akt; pAkt). As shown in Fig. 7d, *Areg* deficiency coincides with reduced levels of pAkt after ischemia and reperfusion. Reconstitution of *Areg*^*−/−*^ mice with recombinant protein normalized pAkt after ischemia reperfusion (Fig. 7d, lower panel and Fig. 7f). This indicates that myocardial levels of AREG expression correlate with activation of survival kinases—such as pAkt—which provides some insight into a potential mechanism for AREG-dependent cardioprotection.

### Areg treatment reconstitutes Hif2a-deficient mice

**Fig. 8:**
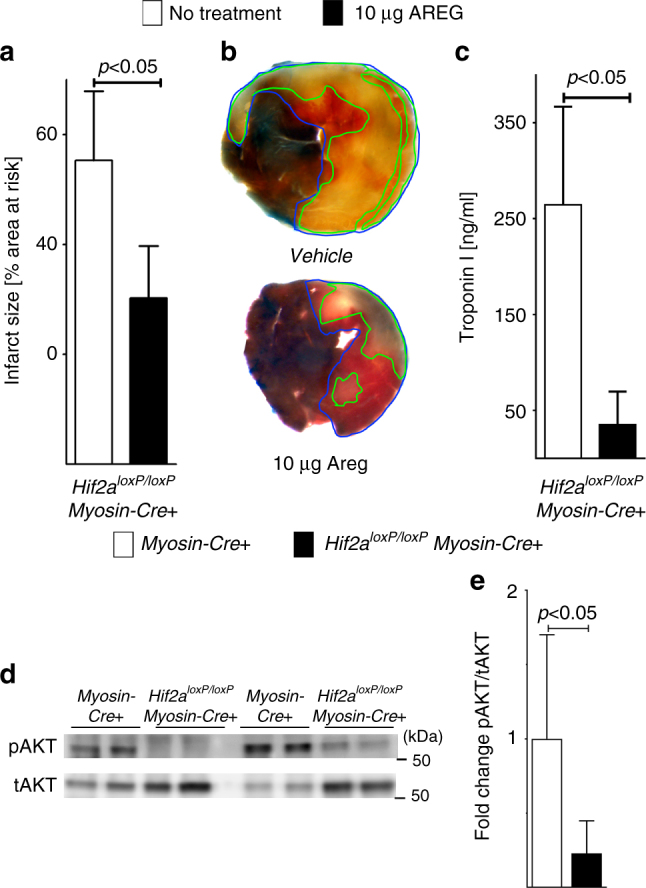
Reconstitution of cardiomyocyte-specific Hif2a-deficient mice with recombinant amphiregulin. **a**–**c** *Hif2a*^*loxP/loxP*^*Myosin-Cre*+ of similar age, gender, and weight as control mice were exposed to 60 min of myocardial ischemia, followed by 120 min of reperfusion; infarct sizes were measured by double staining with Evan’s blue and triphenyltetrazolium chloride and serum samples were collected. All Infarct sizes are presented as the percentage of infarcted tissue in relation to the area-at-risk. Serum troponin levels were determined by ELISA. Note that data in (**a**–**c**) in the “no treatment group” are used in part in Fig. 1c–e to display and analyze IR injury from similar experimental conditions. **a** Infarct sizes of *Hif2a*^*loxP/loxP*^*Myosin-Cre*+ mice after 60 min of ischemia and 120 min reperfusion that were pre-treated with 10 µg recombinant murine AREG administered over a 15 min period via an indwelling arterial catheter or received no pharmacologic intervention. Data presented as the percentage of infarcted area in relation to area-at-risk. Statistical significance assessed by two-sided, unpaired Student’s *t*-test (no treatment *n* = 6; 10 μg AREG *n* = 9, data presented as mean ± SD). **b** Representative infarct staining of *Hif2a*^*loxP/loxP*^*Myosin-Cre*+ that were pre-treated with 10 µg recombinant murine Areg administered over a 15 min period via an indwelling arterial catheter or received no pharmacologic intervention. **c** Serum troponin levels of *Hif2a*^*loxP/loxP*^*Myosin-Cre*+ mice, after 60 min ischemia, 120 min reperfusion that were pre-treated with 10 µg recombinant murine AREG administered over a 15 min period via an indwelling arterial catheter, or received no pharmacologic intervention. Statistical significance assessed by two-sided, unpaired Student’s *t*-test (no treatment *n* = 4; 10 μg AREG *n* = 7, data presented as mean ± SD). **d** *Hif2a*^*loxP/loxP*^*Myosin-Cre*+ or *Myosin-Cre*+ mice underwent 60 min myocardial ischemia, followed by 120 min of reperfusion. Then the area-at-risk was excised, protein isolated and immunoblotted for total AKT (tAKT) or phosphorylated Akt (pAKT). Two samples for each condition and genotype are presented. One representative blot out of three experiments is shown. **e** Quantification by densitometry of pAKT immunoblot results relative to total AKT (tAKT). Data are expressed as mean fold change ± SD normalized to *Myosin-Cre*+. Statistical significance assessed by two-sided, unpaired Student’s *t*-test (three individual blots analyzed with *n* = 7 mice per group)

Next, we investigated whether reconstitution with recombinant Areg rescues the phenotype of mice with cardiomyocyte-specific *Hif2a* deficiency. For this purpose, we treated *Hif2a*^*loxP/loxP*^ *Myosin-Cre*+ mice with 10 µg recombinant Areg given as a slow infusion via a catheter placed into the carotid artery 15 min prior to the onset of myocardial ischemia. Myocardial injury and serum troponin levels after subsequent exposure to 60 min ischemia and 120 min of reperfusion was significantly reduced in comparison to control mice (Fig. 8a–c).

Based on the above studies showing that *Areg* deficiency is associated with reduced activation of the survival kinase Akt after myocardial ischemia and reperfusion, we harvested the AAR form *Hif2a*^*loxP/loxP*^ *Myosin-Cre+* and immunoblotted the isolated protein for total levels Akt and phosphorylated Akt. Consistent with our findings in *Areg*^*−/−*^ mice, we observed that *Hif2a* deficiency in cardiomyocytes is associated with attenuated Akt-phosphorylation in response to ischemia and reperfusion (Fig. 8d, e; Supplementary Fig. 6). In line with previous studies showing cardioprotection via Akt-phosphorylation^33^, these findings indicate the possibility that Hif2-stabilization could mediate cardioprotection via increased activation of Akt through elevating cardiac Areg signaling. Taken together, the above studies indicate that treatment with recombinant Areg is associated with normalized susceptibility of *Hif2a*^*loxP/loxP*^ *Myosin-Cre+* mice to ischemic myocardial injury and provide further evidence for a Hif2-alpha-dependent Areg induction in cardioprotection.

## Discussion

In the present studies, we address the functional roles of HIFs in mediating cardioprotective responses through the induction of specific gene products. As mice with global deletion of HIFs are not viable^34–36^, we generated mice with inducible deletion of *Hif1a* or *Hif2a* in cardiac myocytes. Ischemic tissue injury was similar in *Myosin-Cre*+ and *Hif1a*^*loxP/loxP*^*Myosin-Cre*+ mice, while we uncovered a profound phenotype in *Hif2a*^*loxP/loxP*^*Myosin-Cre*+ mice, including an almost 64% increase of infarct sizes and dramatically elevated serum troponin levels. These data indicate a surprising role for myocyte-specific *Hif2a* in cardioprotection from ischemia. In order to identify potential target genes that could mediate the cardioprotective role of *Hif2a*, we performed a microarray study comparing ischemic cardiac tissues from controls or mice with cardiac myocyte-specific deletion of *Hif2a*. The dominant read-out of this array and confirmatory studies identified an *Hif2a-*specific induction of the epidermal growth factor *Areg* during myocardial ischemia. Indeed, AREG expression was also elevated in cardiac tissues samples of patients with ischemic heart disease. In addition, pharmacologic studies utilizing inhibitors of AREG signaling (AG1478 or cetuximab treatment) showed increased myocardial injury. Moreover, myocardial tissue injury increased in mice with genetic deletion of *Areg*, while treatment of wild-type mice with recombinant Areg attenuated myocardial tissue injury. Finally, reconstitution of *Hif2a*^*loxP/loxP*^ *Myosin-Cre+* mice with recombinant Areg rescued their phenotype. Based on these findings we conclude that HIF2-alpha coordinates the induction of AREG in cardiac myocytes, and thereby conveys potent cardioprotection from myocardial ischemia and reperfusion injury.

In line with the present studies, others have shown a functional role of HIFs in cardioprotection during ischemia and reperfusion injury. For example, inhibition of HIF-degradation provides strong protection from ischemia and reperfusion injury by various mechanisms^37,38^. One study examined the functional role of prolyl-hydroxylase enzymes (PHD), which control the stabilization of HIFs^39^. In this study the authors demonstrated that *Phd1*-deficient mice experienced higher levels of Hif1-alpha and concomitant cardioprotection from ischemia and reperfusion injury. Furthermore, loss of function of PHD2 protects from ischemia and reperfusion injury^40^ and improves cardiac function weeks after ischemia^41^ due to an increase of Hif1-alpha and Hif2-alpha.

Cardioprotective strategies, such as remote preconditioning and ischemic preconditioning, also rely on a stabilization of HIF1-alpha^12,13^. Interestingly, in the present study we show that though HIF1-alpha activity is induced by a single ischemia reperfusion episode (without preconditioning), this activation is not enough to promote cardioprotection. Therefore, HIF1-alpha only confers cardioprotection in ischemic preconditioning settings but not after a single episode of ischemia and reperfusion. This could be related to temporal or tissue-specific issues. For example, it is conceivable that HIF1-alpha stabilization to provide cardioprotection may only be efficient when it occurs prior to the insult. Alternatively, it is conceivable that HIF1-alpha stabilization may occur in the whole heart—particularly the myocyte compartment—however, HIF1-alpha-dependent cardioprotection through ischemic preconditioning may involve different tissue compartments of the heart—such as the myeloid compartment or endothelial cell. Interestingly, a recent study found that vascular-endothelial *Hif1a* mediates the acute phase of cardioprotection by ischemic preconditioning^42^, whereas our previous study suggest that HIF1-alpha is essential for the protective effects of ischemic preconditioning^14^. In contrast to this, ischemic preconditioning was preserved in mice with cardiomyocyte-specific deletion of HIF2-alpha. This suggests that the axis HIF2-alpha-AREG does not play a role in ischemic preconditioning. Pharmacologic HIF activators can be used to imitate the cardioprotective responses elicited by ischemic preconditioning^11^. In contrast to this, HIF1-alpha in cardiomyocytes does not influence myocardial injury in ischemia and reperfusion as shown in the present study. This indicates that myocyte-mediated cardioprotection through HIFs predominantly involves HIF2-alpha. Thus, the present findings suggest that myocyte-specific HIF2-alpha increases myocardial ischemia tolerance, which has not been previously reported. Along these lines, studies in other organs than the heart have implicated HIF2-alpha in organ protection from ischemia and reperfusion injury, for example during ischemia and reperfusion injury of the kidney^17^.

Our findings implicate myocyte-specific *Hif2a* in cardioprotection from ischemia and reperfusion injury, whereas previous studies have found a role for *Hif1a* in mediating cardioprotection elicited by ischemic preconditioning^11,12^. Indeed, based on several previous studies, it appears that HIF1-alpha is essential for cardiac ischemic preconditioning.^11,14^ At first look, these findings appear to be in contrast with the present studies showing a cardioprotective role for HIF2-alpha as well. However, different protocols (ischemic preconditioning versus ischemia and reperfusion injury) and different tissue compartments (e.g. vascular compartment^42^ versus the myocyte compartment^14^) likely account for functional differences of the HIF isoforms HIF1-alpha and HIF2-alpha. Specifically, a previous study from the laboratory of Dr. Semenza demonstrated that mice with heterozygote deletion of *Hif1a* are not protected by ischemic preconditioning^12^. Similarly, other studies indicate that *Hif1a* repression with siRNA prevents cardioprotection by ischemic preconditioning. However, the role of HIF2-alpha in cardioprotection has previously been unclear. To address the functional role of *Hif2a* during myocardial ischemia and reperfusion injury, we used genetic models and found that myocyte-specific *Hif2a* is critical for the cardioprotection during ischemia and reperfusion injury. To address the functional role of *Hif2a* in ischemic preconditioning we performed additional studies. Here we found that cardioprotection by ischemic preconditioning is intact in control mice (Myosin-Cre+ mice) or *Hif2a*^*loxP/loxP*^*Myosin-Cre*+ mice, whereas cardioprotection by ischemic preconditioning is abolished in *Hif1a*^*loxP/loxP*^*Myosin-Cre*+ mice (Supplementary Fig. 7A–C). Together, with previous studies, these findings indicate a somewhat specific role for HIF1-alpha in mediating cardioprotective effects of ischemic preconditioning^11,42^.

Previous studies on the role of HIF1-alpha found that the cardioprotective effects of ischemic preconditioning can be pharmacologically recapitulated utilizing the HIF activator DMOG. Importantly, the PHD inhibitor DMOG will result in the stabilization of both HIF isoforms—HIF1-alpha and HIF2-alpha—in different cardiac tissue compartments, including the vasculature and cardiac myocytes. To answer the question if DMOG-elicited cardioprotection also involves HIF2-alpha, we performed additional studies. Here, we found that the cardioprotective effect of DMOG is intact in control mice (Myosin-Cre+ mice) but attenuated in mice with myocyte-specific deletion of Hif2a (*Hif2a*^*loxP/loxP*^*Myosin-Cre*+ mice; Supplementary Figure 8A, B). Similarly, in mice with siRNA-mediated *Hif1a* repression, DMOG-dependent cardioprotection was attenuated, as we had reported previously^11^. Together, these findings indicate that DMOG likely mediates cardioprotection via stabilization of both HIF isoforms—HIF1-alpha and HIF2-alpha. Previous studies suggest that HIF1-alpha-dependent cardioprotection involves purinergic signaling events, particularly through the A2B adenosine receptor^11,14^, whereas the current studies implicate AREG signaling as a mediator of HIF2A-dependent cardioprotection. This notion is also supported by the current findings that *Hif2a*^*loxP/loxP*^*Myosin-Cre*+ mice can be rescued via treatment with recombinant AREG.

The present finding that HIF2-alpha regulates AREG functionally is in line with previous studies investigating the transcriptional pathway of AREG induction during hypoxic conditions. For example, a previous microarray analysis of colon-derived epithelial cells revealed a hypoxia-dependent increase in AREG expression^23^. Similarly, the authors of this study found that AREG expression was also induced in tissues from mice exposed to whole animal hypoxia. Interestingly, and consistent with the present findings from myocardial ischemia and reperfusion injury, this study revealed that AREG induction was independent of the classic transcriptional response mediated via HIF1-alpha, but rather indicated an evolutionarily conserved cAMP response element (CRE) that constitutively binds the CRE binding protein (CREB) in AREG induction during hypoxia^23^. More recent studies from the cancer field provide direct evidence for a functional role of HIF2-alpha in the induction of AREG expression during conditions of hypoxia via direct binding of HIF2-alpha to the *AREG* promoter, and thereby increasing its transcriptional activity^22,43^. These studies found that the PHD-HIF2A-AREG pathway influences breast cancer progression and suggest PHD2 as a potential tumor suppressor in breast cancer^43^. However, the functional role of HIF2-alpha-dependent induction of AREG in myocardial ischemia and reperfusion injury has not been previously examined. Indeed, our findings implicate the PHD–HIF2A–AREG pathway in mediating increased ischemia tolerance of the myocardium.

The present studies provide pharmacologic evidence that AREG-mediated cardioprotection involves the growth factor receptor ERBB1 receptor. Specific inhibition^28^ for the AREG receptor ERBB1 produced a similar phenotype in mice as genetic deletion of *Areg*. Consistent with these findings, previous studies had demonstrated that AREG binds to the ERBB1 receptor at a low affinity, thereby causing constant activation of ERBB1^44^. This distinguishes AREG from other endogenous ligands for the ERBB1 receptor. For example, EGF binds to ERBB1 with high affinity, causing a strong and rapid activation, but it also causes receptor internalization and subsequent degradation, thereby terminating its signaling events^45^. Moreover, several potential mechanisms could influence how the HIF2A–AREG–ErbB1 signaling cascade provides cardioprotection. Previous studies have found a link between ErbB1 signaling and the inhibition of apoptosis via activation of “survival kinases”^46^. For example the ErbB1/ErbB2 heterodimer was found to activate AKT, which in turn blocks p53—a master regulator of apoptosis^47^. In the present study, AREG expression during myocardial ischemia, exogenous AREG administration or *Hif2a* expression, and HIF2-alpha stabilization all increased the activated form of AKT. This suggests that AKT activation in cardiomyocytes upon ischemia–reperfusion is executed by HIF2-alpha and AREG. Indeed, previous studies found a dominant role of AKT activation in ischemic preconditioning^48^. Since *Hif2a*^*loxP/loxP*^*Myosin-Cre*+ undergo ischemic preconditioning, whereas *Hif1a*^*loxP/loxP*^*Myosin-Cre*+ do not, we consider that AKT activation in cardiomyocytes during ischemia preconditioning is driven by HIF1-alpha. Independent of AKT activation, ERBB1 reduces apoptosis via inhibition of caspase-3 and caspase-9 activity^49^. However, other studies found that apoptosis contributes to myocardial injury after days or maybe weeks^50^. In the acute setting, such as used in the present study, necrosis mainly determines infarct sizes and the influence of apoptosis to myocardial injury is considered rather limited^51^. Thus, we believe that in our study, the anti-apoptotic effect of AREG–ERBB1 signaling only has a minor impact on infarct sizes. Other studies have indicated that AREG signaling dampens acute inflammation, such as occurs in the context of myocardial ischemia and reperfusion injury^52,53^. For example in a model of skeletal muscle injury, regulatory T cells (T_regs_) strongly upregulate AREG, while treatment with recombinant AREG enhances muscle repair after injury^54^ via suppressive T_regs_. Other studies suggest that ERBB1 receptor activation promotes the glycolytic capacity, and thereby provide optimization of cellular metabolism during conditions of limited oxygen availability—such as occurs during myocardial ischemia^52,53,55^

In patients with IHD we found a strong induction of AREG protein levels. This is consistent with a previous microarray study showing a 16-fold increase of AREG transcript levels and a 3-fold increase in protein expression in patients undergoing coronary bypass surgery^56^. In view of the present findings, the *AREG* transcript and protein increase during bypass surgery is likely caused by stabilization of HIF2-alpha protein levels, and could reflect an endogenous protective pathway. Indeed, in the present studies, treatment with recombinant AREG provides robust cardioprotection from ischemia and reperfusion injury (see Fig. 7). Together, these findings suggest that pharmacologic HIF activators (particularly for HIF2-alpha) or treatment with recombinant AREG could be used to prophylactically treat cardiac surgery patients to enhance myocardial ischemia tolerance, and thereby improve outcomes of these major surgical interventions. Similarly, patients at risk for myocardial injury could receive such a treatment approach.

Taken together, the present studies identify a previously un-identified role for myocyte-specific HIF2-alpha in protection from myocardial ischemia and reperfusion injury. Extensions of these findings suggest *HIF2A*-dependent induction of AREG in cardioprotection from ischemia, and implicate pharmacologic strategies that stabilize HIFs or promote AREG signaling in cardioprotection from ischemia and reperfusion injury.

## Methods

### Cell culture and hypoxia

HCM were purchased from Sciencell Inc. (Carlsbad, CA). ScienCell Inc. guarantees that cells are free of mycoplasma contamination. Cells were cultured according to the manufacturer’s instructions and then transferred into a humidified hypoxia chamber containing 1% oxygen (Coy LaboratoryProducts).

### Knockdown of HIF1A and HIF2A in vitro

Stable cell cultures with decreased HIF1A and HIF2A expression were generated by lentiviral-mediated shRNA expression. Sigma’s MISSION pLKO.1 lentiviral vectors targeting HIF1A had shRNA sequence of CCGGCCAGTTATGATTGTGAAGTTACTCGAGTAACTTCACAATCATAACT GGTTTTT (TRCN0000003809) and HIF2A had a sequence of CCGGCCATGAGGAGATTCG TGAGAACTCGAGTTCTCACGAATCTCCTCATGGTTTTT (TRCN0000003807). For controls, non-targeting control shRNA (SHC001) was used. HEK293T (American Type Culture Collection^®^ Inc.) cells were co-transfected with pLK0.1 vectors and packaging plasmids to produce lentivirus. Filtered supernatants and 8 µg/ml of Polybrene were used for infection of HCM and cells were selected with puromycin dihydrochloride (Sigma) (2 µg/ml).

### Mice

All animal procedures were performed in an AAALAC-accredited facility in accordance with the Guide for the Care and Use of Laboratory Animals and approved by the University of Colorado Denver Institutional Animal Care and Use Committee. We used male mice with an age of 8- to 16-week-old mice in all studies. All experiments fulfilled the NIH guidelines for use of live animals. To generate cardiac myocytes specific-deletion, *Myosin-Cre*+ (B6.FVB(129)-Tg(Myh6-cre/Esr1*), *Hif1a*^*loxP/loxP*^ (B6.129-*Hif1atm3Rsjo*/J), and *Hif2a*^*loxP/loxP*^ (Epas1tm1Mcs/J) were purchased from Jackson Laboratory (Bar Harbor, ME) and crossbred. To induce Cre-recombinase activity, mice underwent tamoxifen treatment for five consecutive days with 1 mg i.p. per day. Tamoxifen was dissolved in sterile peanut oil and administered in a volume of 100 μl. Between the last tamoxifen injection and experimentation were at least 7 days for recovery.

*Areg*^*−/−*^ (B6;129-Areg^tm1Dle^/Mmnc)^24^ mice were purchased from Mutant Mouse Regional Resources Centers (Chapel Hill, NC). *Areg*^*−/−*^ mice were maintained using heterozygouse breeding strategy (*Areg*^*+/−*^) to bypass lactation difficulties in younger females. As controls, we used mice with the same genetic background as *Areg*^*−/−*^ (B6,129) mice. Genotyping in all strains used was performed by GeneTyper Inc. (NY, USA).

### Murine model of myocardial ischemia

Mice underwent in situ myocardial ischemia and reperfusion injury as described previously^57,58^. In short, we used sodium pentobarbital to induce and maintain anesthesia. For induction we administered sodium pentobarbital in a dose of 70 mg/kg i.p., to maintain anesthesia in a dose of 10 mg/kg/h i.p.. The mice rested on a temperature-controlled heated table (RT, Effenberg, Munich, Germany) and were mechanically ventilated with a pressure-controlled ventilation mode (ventilator settings: inspiratory pressure 10 mbar, frequency 110 breaths/min, positive end-expiratory pressure of 5 mbar, FiO_2_ = 0.4). After thoracotomy, we visualized the left coronary artery (LCA), placed an 8.0 nylon suture (Prolene, Ethicon, Norderstedt, Germany) around the vessel, and connected hanging weights to each end of the suture to occlude the LCA. We confirmed successful occlusion by color change of the vessel (from light red to dark violet). In addition, the coloration of the myocardium supplied by the LCA changed from bright red to pale and an ST-elevation on the connected EKG was visible. After 60 min of ischemia, the weights were taken off and reperfusion occurred. This was confirmed by reversal of the color changes of vessel and tissue described above. In a subset of experiments, mice underwent ischemic preconditioning as described previously^59^. In short, ischemic preconditioning represents an experimental strategy, where short non-lethal episodes of myocardial ischemia result in cardioprotection. Surgical preparation was performed as described above. Subsequently, we performed four cycles of 5 min of ischemia, followed by 5 min of reperfusion, before 60 min of ischemia and 120 min of reperfusion were initiated. If the investigator performing the mouse experiment observed procedural abnormalities (e.g. incomplete ischemia due to collateral circulation, involuntary ischemic preconditioning), animals were excluded from analysis. The investigator was not blinded to genotype and treatment. After 120 min of reperfusion, we determined the infarct sizes by calculating the percentage of myocardium that underwent infarction compared to the AAR using the double staining technique with Evan’s blue and triphenyltetrazolium chloride (TTC). Here, we permanently occluded the suture, followed by injection of Evan’s blue until the dye was visible in the cardiac veins. The heart was then excised and sliced into eight slices, 0.5 mm thickness each using a heart matrix (Roboz, Inc.). The slices were incubated in a TTC at 37 °C for 15 min and then transferred into formalin for fixation overnight. One day after the experiment, the heart slices were put between a histology slide, with a clamp on each side and photograph with a digital camera. Then, AR and the infarct size were determined by planimetry using the ImageJ (National Institutes of Health). The infarct sizes were determined, using the following formula: infarct size = sizes of infarct/size area-at-risk × 100. We have previously described the details of this technique^58,59^.

### Microarray analysis

*Myosin-Cre*+ and *Hif2a*^*loxP/loxP*^*Myosin-Cre*+ were used for microarray analysis to profile the transcriptional response 45 min ischemia and 120 min reperfusion. We used untreated littermates of the same genotype as baseline control animals. Unpooled RNA, isolated from heart tissue using four of four animals per group, was profiled on Agilent-014868 Whole Mouse Genome Microarray 4x44K arrays (G4122F; Agilent Technologies, Palo Alto). Array data were normalized using quantile normalization and statistical analysis performed using two-factor ANOVA (treatment and genotype) in the Partek Genomics Suite (Partek Inc., St Louis, MO). Benjamini–Hochberg false discovery rate (FDR) was used to control for multiple comparisons. Molecules from the dataset that met the fold change cutoff of 2 and 5% FDR were included in the subsequent analysis. Myocardial ischemia–reperfusion largely influenced the gene expression in both genotypes. To identify *Hif2a*-regulated genes, we focused on the difference between the two genotypes after myocardial IR injury using the formula: [(*Myosin-Cre+*_myocardial IR_ −* Myosin-Cre+*_myocardial Control_) – (*Hif2a*^*loxP/loxP*^*Myosin-Cre*+_myocardial IR_ − *Hif2a*^*loxP/loxP*^*Myosin-Cre*+_Control_)]. Fisher’s exact test was used to calculate a *p*-value determining the probability that the association between the genes in the dataset is explained by chance alone. Array data have been deposited at http://www.ncbi.nlm.nih.gov/geo/ (accession number GSE67308).

### Human cardiac tissues

In proof of principle studies, AREG protein levels were measured in human cardiac tissues. Collection and use of these samples was approved by the Colorado Multiple Institutional Review Board (COMIRB). Prior to use, the collecting institution (Division of Cardiology, Department of Medicine, University of Colorado Denver, Aurora, Colorado, USA) de-identified all heart samples. We obtained these, via the “University of Colorado–Division of Cardiology Biobank”^14^. Control cardiac tissues were derived from the left ventricle of healthy hearts considered for transplantation, but could not be transplanted into a recipient due to logistic or other reasons. In case of the nonfailing donor hearts, the family/next of kin gave permission for organ/tissue donation for purposes of transplantation and research. Ischemic cardiac tissue was obtained from the left ventricle of patients with ischemic cardiomyopathy, in whom the hearts were explanted during the process of orthotropic heart transplantation.

### Transcriptional analysis

Primer set (sense sequence and antisense sequence, respectively) for murine Amphiregulin was as following: *Areg* (Qiagen, Cat# QT00112217); in case of human cell samples the following primer sets were used: *AREG* (Qiagen, Cat# QT02450469); *HIF1A* (Qiagen, Cat# QT00083664); *HIF2A* (Qiagen, Cat# QT00069587). Each target sequence was amplified using increasing numbers of cycles of 94 °C for 1 min, 58 °C for 0.5 min, 72 °C for 1 min. β-actin mRNA (Murine: Qiagen Cat# QT00095242; human: Qiagen, Cat# QT01680476) was amplified in identical reactions to control for the amount of starting template. Levels and fold change in mRNA were determined using the *Pfaffl *method^60^.

### Immunoblotting experiments

To measure Hif1a, Hif2a, Areg, total AKT, or phosphorylated AKT protein content in the post-ischemic murine myocardium, we euthanized the animals after 60 min of myocardial ischemia and 120 min reperfusion. Subsequently, we flushed the circulation via an arterial catheter with ice-cold normal saline solution, identified the myocardial tissue that underwent ischemia and immediately shock frozen this section of the heart in liquid nitrogen. For immunoblotting studies, murine or human tissues samples were homogenized and lysed for 10 min in ice-cold lysis buffer (150 mM NaCl, 25 mM Tris, pH 8.0, 5 mM EDTA, 2% Triton X-100, and 10% mammalian tissue protease inhibitor cocktail; Sigma-Aldrich). For immunoblotting studies in HCM, we lysed the cells in 5× PAGE buffer, centrifuged the cell suspension at 14,000*g* to remove cell debris, and discarded the pellet. Proteins were solubilized in reducing Laemmli sample buffer and heated to 90 °C for 5 min. Samples were subsequently resolved on a 12% polyacrylamide gel and transferred to nitrocellulose membranes. The membranes were blocked for 1 h at room temperature in phosphate-buffered saline (PBS) supplemented with 0.2% Tween 20 and 4% bovine serum albumin. The antibody to detect human HIF1a was purchased BD transduction laboratories (clone 54/HIF-1a; catalog-# 610958). From Novus Biologicals we purchased antibodies to detect murine Hif1a (clone H1alpha67; catalog-# NB100-105) or Hif2a (clone ep190b; catalog-# NB100-132). Antibodies were purchased from Santa Cruz Biotechnology to blot for human AREG or murine Areg (clone G-4; catalog-# sc-74501). From Cell Signal Technology, we purchased an antibody to detect total Akt (clone 40D4; catalog-# 2920) and phosphorylated Akt (clone D25E6; catalog-# 13038). The anti-actin antibody (clone JLA20; catalog-# CP01) was purchased from EMD Millipore. The membranes were incubated with the primary antibody in 10 μg/ml (dilution 1:1000) overnight at room temperature, followed by 10-min washes in PBS. The membranes were then incubated in 1:5000 goat anti-mouse horseradish peroxidase. The wash was repeated, and proteins were detected by enhanced chemiluminescence. Densitometry was performed using ImageJ 1.51J8 as described^61^. Full gel scans of all western blots presented in the figure can be found in the supplementary figures (Supplementary Figs. 1–6).

### Recombinant murine Areg treatment

Carrier-free, recombinant mouse Amphiregulin protein (Areg) was purchased from R&D Systems (Minneapolis, MN). In short, Amphiregulin was dissolved in 0.9% NaCl solution and 10 µg were administered over 15 min by a syringe pump connected to an indwelling carotid artery catheter. When the injection was complete, we began the in situ myocardial ischemia.

### AG1478 treatment

The ErbB1 inhibitor AG1478 was purchased from Tocris (Bristol, United Kingdom). AG1478 was dissolved in 100 mM Captisol (Cydex, Overland Park, KS) at a concentration of 5 mM as described^62^. Prior myocardial ischemia, we administered 200  mmol/l/kg AG1478 over a 15 min period via the carotid artery catheter connected to a syringe pump^28^. Then, we continued with our myocardial ischemia model.

### Cetuximab treatment

Cetuximab (Erbitux^®^) was purchased from Bristol-Myers Squibb. This monoclonal antibody is specifically directed against the extracellular domain of ErbB1, thereby completely blocking signaling through this receptor^30^. Immediately prior to myocardial ischemia, we diluted cetuximab in a sterile, isotonic sodium chloride solution and administered 20 mg/kg over a 15 min period via the carotid artery catheter connected to a syringe pump^28^. Then, we continued with myocardial ischemia and reperfusion injury as described above.

### Dimethyloxaloylglycine treatment

Dimethyloxaloylglycine (DMOG) was purchased from Sigma-Aldrich. DMOG permeates the cells and inhibits this prolyl-4-hydroxylase, which upregulates HIFs. As described previously^11^, mice received 1 mg DMOG via a single intraperitoneal injection 4 h prior to myocardial ischemia and reperfusion injury—as described above.

### Data analysis

Based on previous studies, we performed an a priori sample size analysis for infarct sizes (standard deviation, SD, 5%) and serum troponin measurements (SD 21 ng/ml)^57^. We consider a difference biologically relevant, when infarct sizes differ at least 10% or serum troponin levels at least 45 ng/ml between control and experimental groups. Based on this, we estimated that we required at least five animals per group to detect a statistical significant difference with a power of 80%. All data are presented as mean ± SD. To test for statistical significant differences between two groups, we used the unpaired Student’s *t-*test. To compare more than two groups, one-way analysis of variance (ANOVA) with Bonferroni’s multiple comparison test was used; per journal policy, the degree of freedom and the F-value are listed in the figure legends. All comparisons in the current study underwent two-tailed testing and we considered *p* < 0.05 to be significant. For samples sizes, outlier analysis (ROUT method) and statistical analyses we used StatMate^®^ and Prism^®^ (both GraphPad Software Inc.). Statistical analysis was reviewed by a professional UT Health statistician prior to submission. Sample sizes are provided in the figure legends. All authors had full access to and take full responsibility for the integrity of the data. All authors have read and agree to the manuscript as written.

### Data availability

The array dataset was deposited in GEO under accession code GSE67308. All relevant data are available from the corresponding author upon reasonable request.

## Electronic supplementary material

Supplementary information
