# Supplementary material for: Hypoxia-inducible factor 2-alpha-dependent induction of amphiregulin dampens myocardial ischemia-reperfusion injury
Source: Nat Commun. 2018 Feb 26;9:816. doi: 10.1038/s41467-018-03105-2 (PMC5827027; doi:10.1038/s41467-018-03105-2)
Supplement: Supplementary file 1 — Supplementary information [file 41467_2018_3105_MOESM1_ESM.pdf]

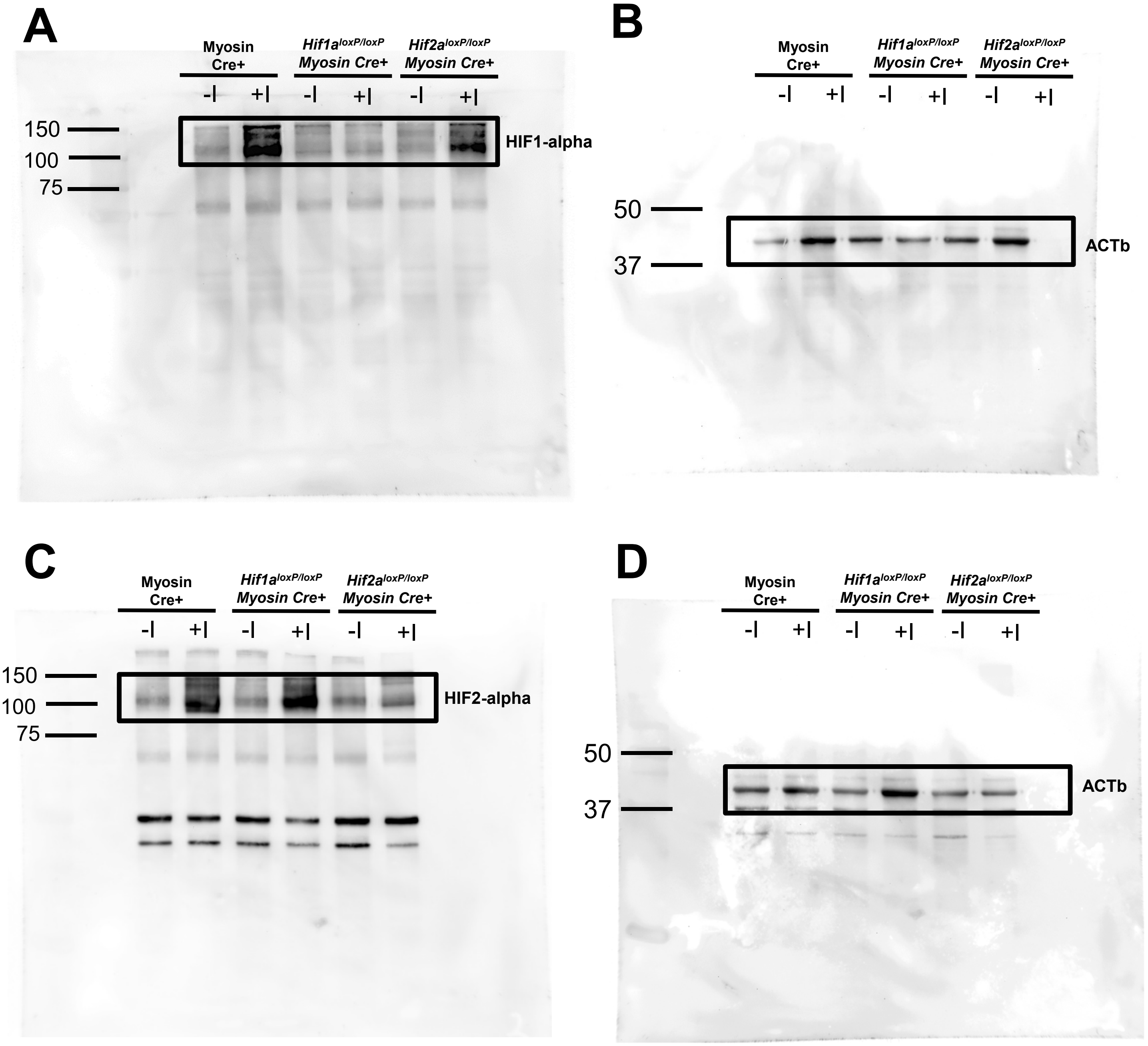

Supplementary figure 1. Full gel scans for Fig. 1B (A+B) Gel scans for figure 1B; HIF1-alpha blot with corresponding beta-Actin blot; **(C+D)** Gel scans for figure 1B; HIF2-alpha blot with corresponding beta-Actin blot;

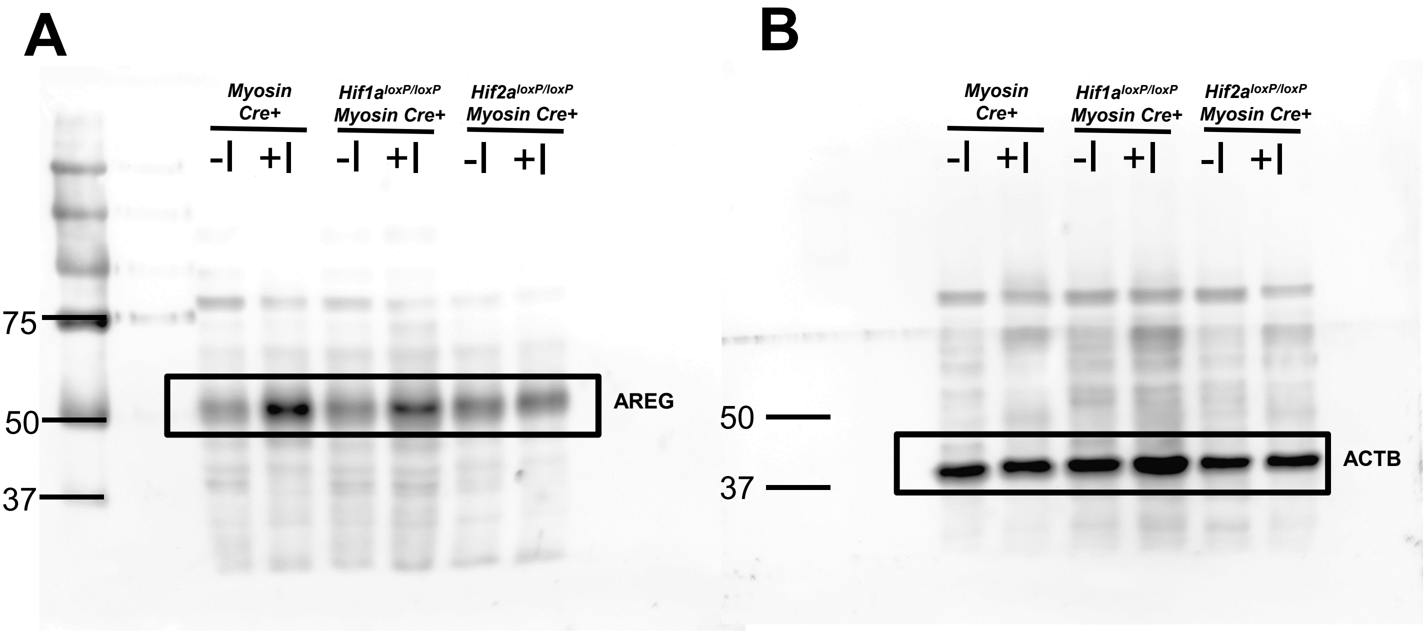

Supplementary figure 2. Full gel scans for Fig. 2D (A+B) Gel scans for figure 1B; AREG blot with corresponding beta-Actin blot;

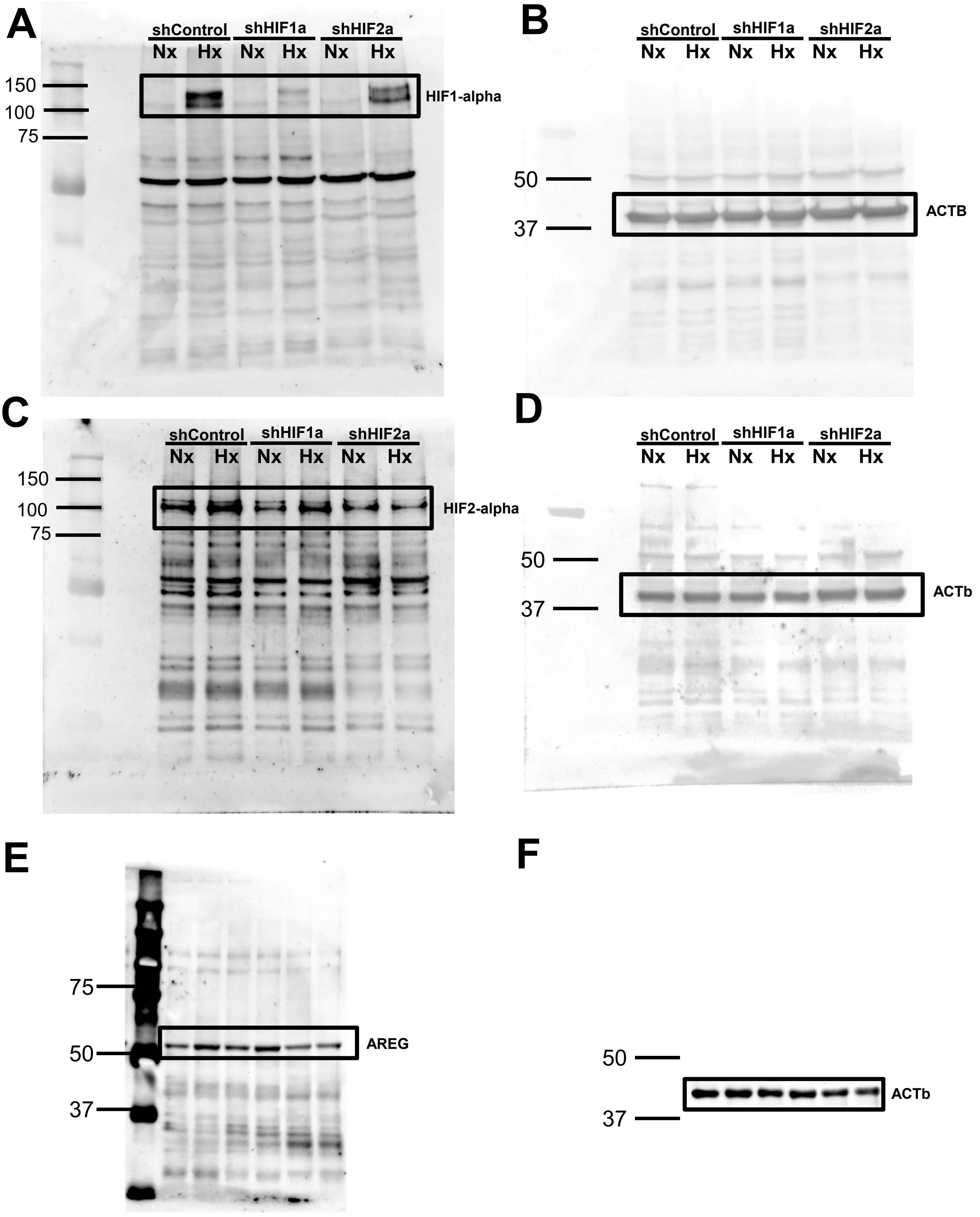

Supplementary figure 3. Full gel scans for Fig. 3D+G (A+B) Gel scans for figure 3C; HIF1-alpha blot with corresponding beta-Actin blot; **(C+D)** Gel scans for figure 3C; HIF2-alpha blot with corresponding beta-Actin blot; **(E+F)** Gel scans for figure 3G; AREG blot with corresponding beta-Actin blot;

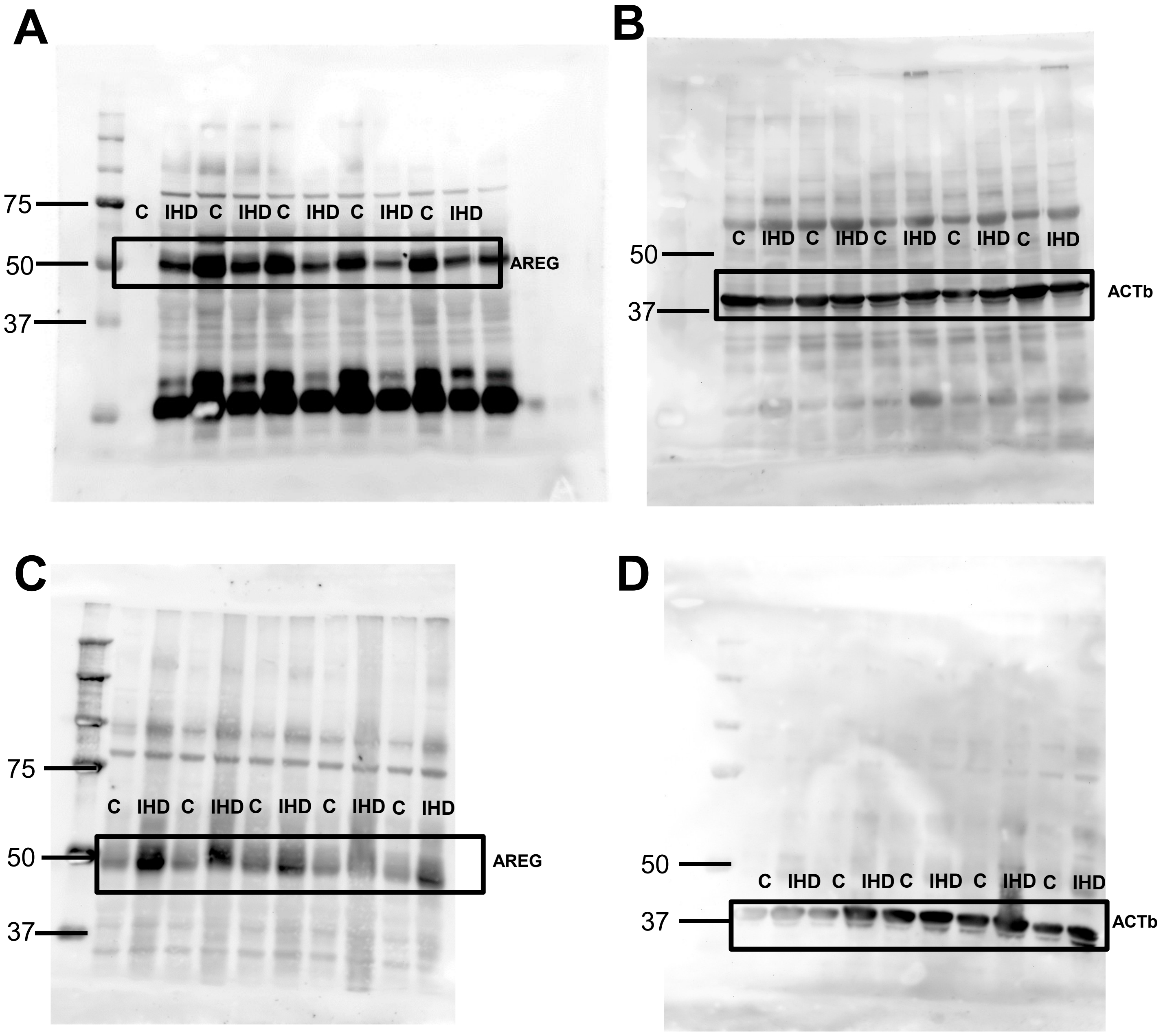

Supplementary figure 4. Full gel scans for Fig. 4A. Gel scans for figure 4A; AREG blot with corresponding beta-Actin blot;

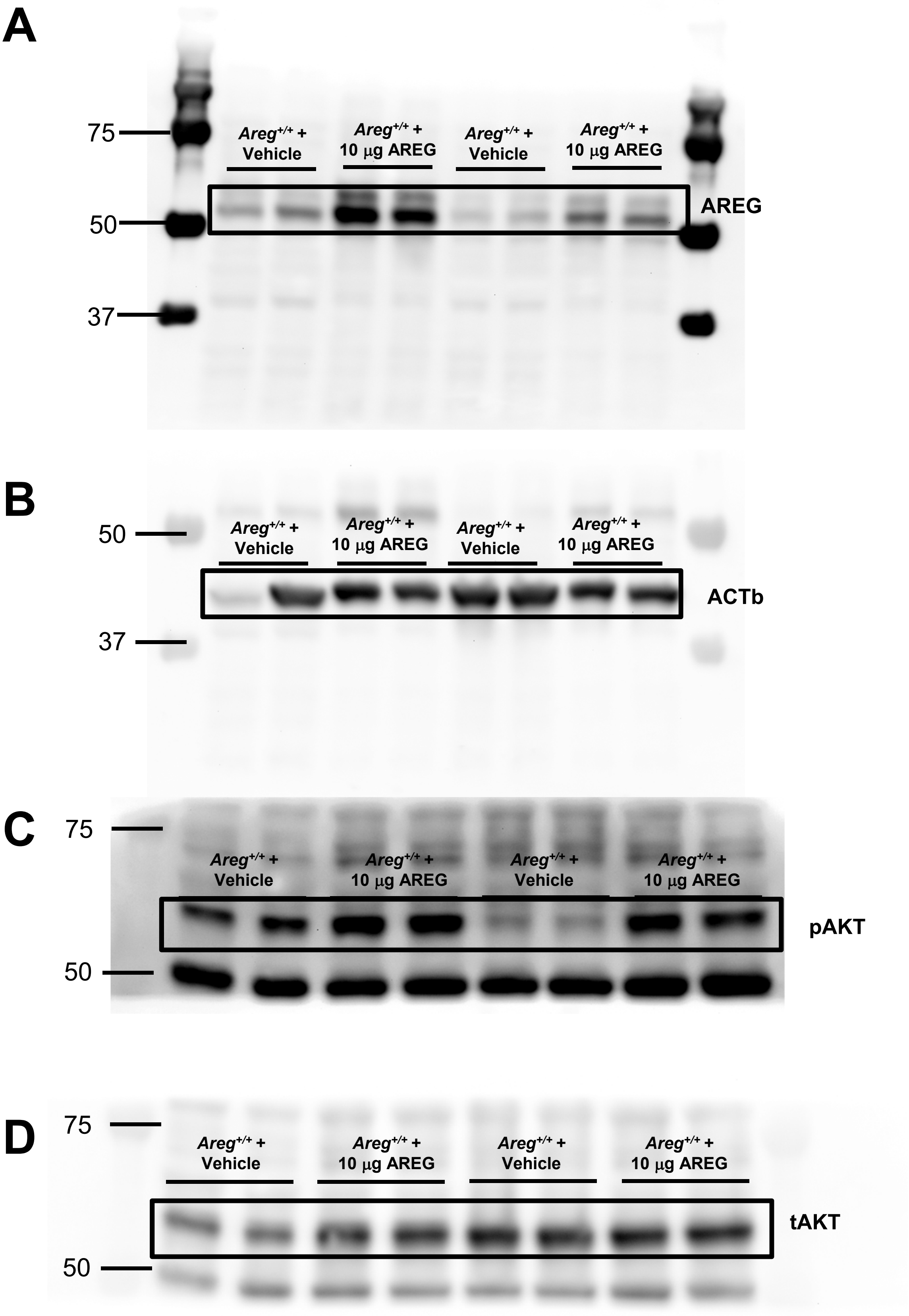

Supplementary figure 5. Full gel scans for Fig. 7A.; **(A+B)** AREG blot with corresponding beta-Actin blot in mice treated with vehicle or 10 µg recombinant murine AREG 30 min prior to ischemia induction. **(D+E)** pAKT and corresponding tAKT blot

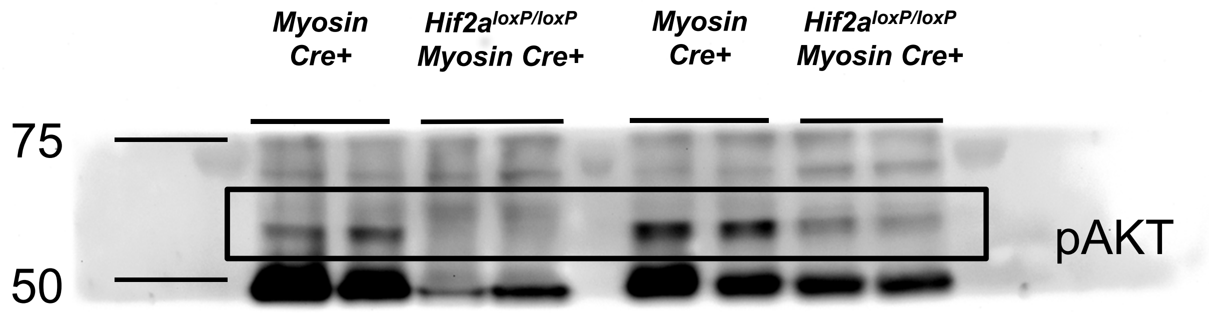

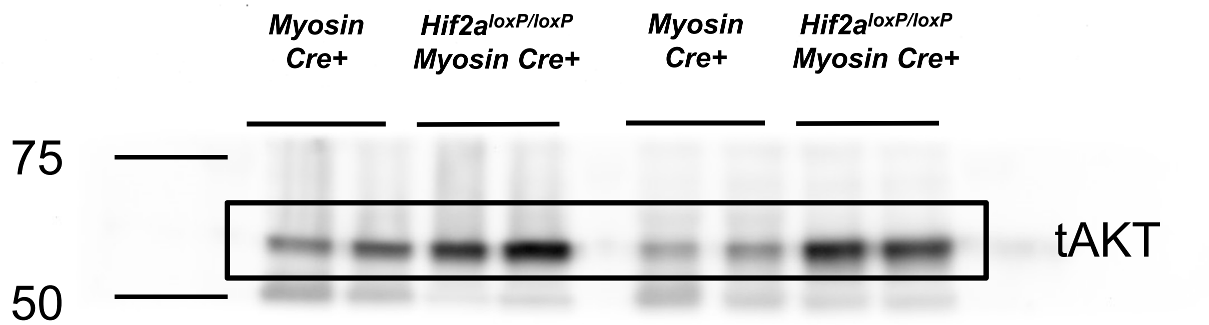

Supplementary figure 6. Full gel scans for Fig. 8D. (A) pAKT blot with **(B)** corresponding total-Actin blot;

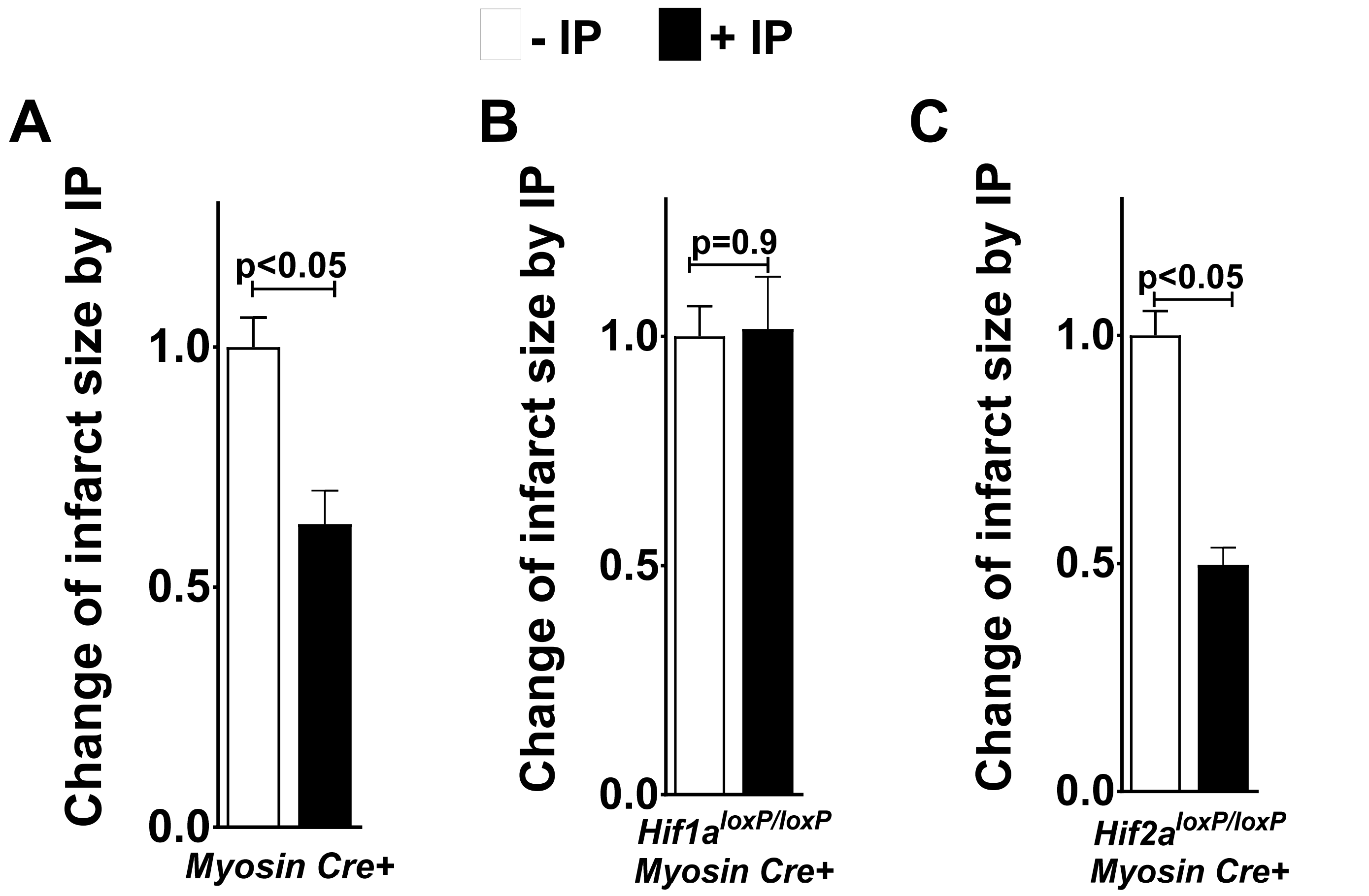

Supplementary figure 7. ***Contribution of myocyte-specific hypoxia-inducible factor (HIF) isoforms Hif1a or Hif2a to cardioprotection by ischemic preconditioning.*** **(A-C)** *Myosin-Cre+, Hif1a$^{loxP/loxP}$ Myosin-Cre+ or Hif2a$^{loxP/loxP}$ Myosin-Cre+* were exposed to 60 min of myocardial ischemia with ischemic preconditioning (+IP; four cycles of 5 min of ischemia followed by 5 min of reperfusion) or without IP (−IP) followed by 120 min of reperfusion; infarct sizes were measured by double staining with Evan's blue and triphenyltetrazolium chloride and serum samples were collected. Infarct sizes were determined as percentage to the area-at-risk and then normalized to –IP group of the respective genotype. ***Note that data in (A)-(C) are used in Fig. 1C-E to display and analyze IR injury from similar experimental conditions.*** **(A)-(C)** Infarct sizes of +IP relative to –IP group of **(A)** *Myosin-Cre+* (n=4 per group) *,* **(B)** *Hif1a$^{loxP/loxP}$ Myosin-Cre+* (n=4 per group) or **(C)** *Hif2a$^{loxP/loxP}$ Myosin-Cre+* (n=4 per group). All data presented as mean ± SD. Statistical significance was assessed by two-sided, unpaired Student's t-test.

Note: Consistent with previous studies showing a central role of HIF1A in cardio-protection mediated by IP treatment, IP-protection is selectively abolished in Hif1a$^{loxP/loxP}$ Myosin-Cre+ but remains intact in Myosin-Cre+ or Hif2a$^{loxP/loxP}$ Myosin-Cre+.

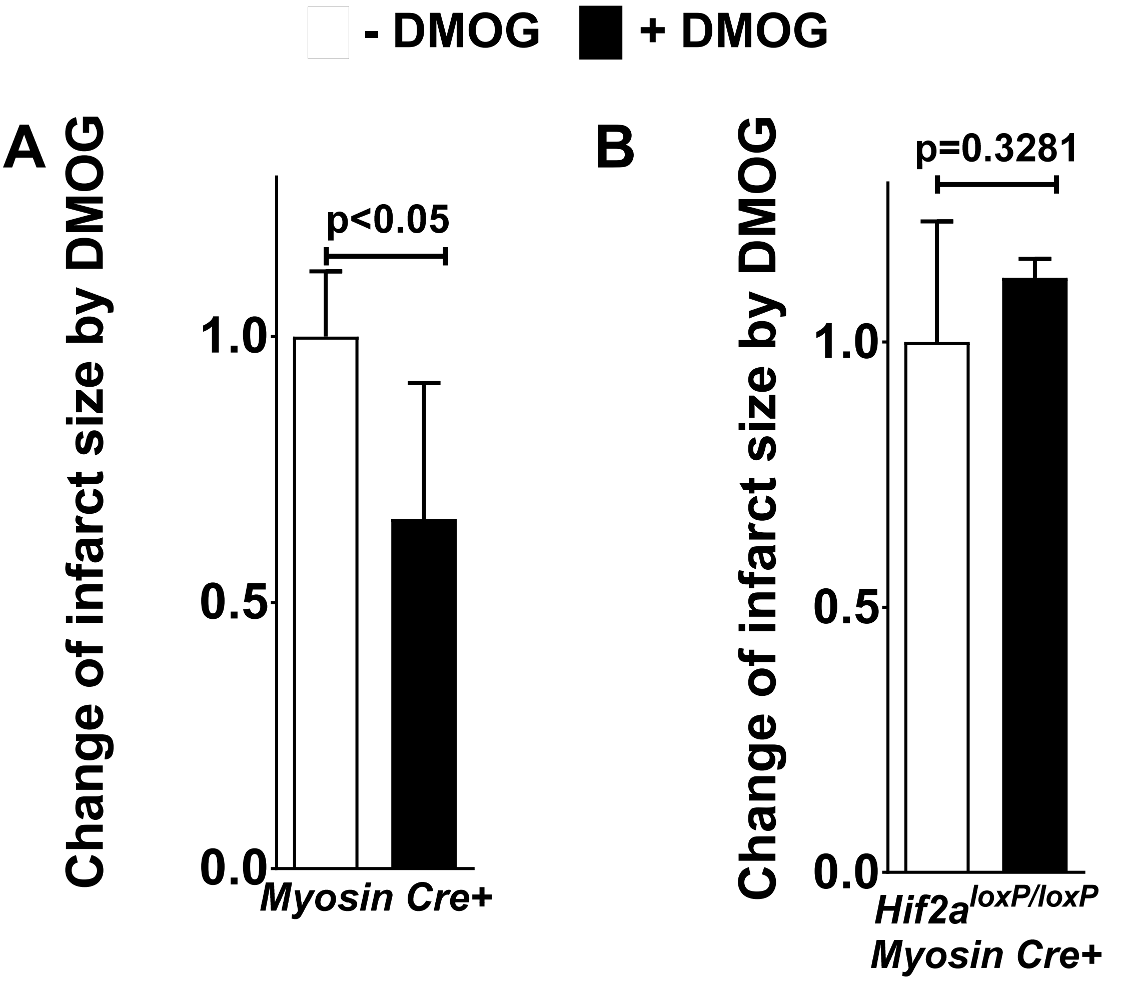

Supplementary figure 8. Role of myosin-specific Hif2a in the cardioprotection mediated by the pharmacologic HIF activator dimethyloxaloylglycine (DMOG)*.* (A, B) *Myosin-Cre+* mice were treated with an intraperitoneal injection of vehicle or 1 mg DMOG four hours before they underwent myocardial ischemia and reperfusion. For this purpose they were exposed to 60 min of myocardial ischemia followed by 120 min of reperfusion; infarct sizes were measured by double staining with Evan's blue and triphenyltetrazolium chloride. Infarct sizes were determined as percentage to the area-at-risk, followed by normalization to the –DMOG group. n=4 per group. All data presented as mean ± SD. Statistical significance was assessed by two-sided, unpaired Student's t-test.
